# Leadership and Organizational Responses After Patient Safety Incidents: A Systematic Review of Second-Victim Outcomes and Evidence Gaps in Safety Behaviors

**DOI:** 10.3390/healthcare14183002

**Published:** 2026-09-14

**Authors:** Georgia Kyriakeli, Iro Tsara, Eleni Lazaridou, Agapi Symeonidou, Zoi Tsimtsiou, Vasilios Kotsis

**Affiliations:** 1 Nursing Service, General Hospital Papageorgiou, 56429 Thessaloniki, Greece; irotsara@yahoo.com (I.T.); elenlazaridou83@gmail.com (E.L.); solovetha@gmail.com (A.S.); 2 Department of Hygiene, Social-Preventive and Medical Statistics, School of Medicine, Aristotle University of Thessaloniki, 54124 Thessaloniki, Greece; ztsimtsiou@auth.gr; 3 3rd Department of Internal Medicine, School of Medicine, Aristotle University of Thessaloniki, 54124 Thessaloniki, Greece; vkotsis@auth.gr

**Keywords:** second victim, patient safety incidents, leadership, organizational support, just culture, patient safety culture, peer support, healthcare professionals

## Abstract

**Highlights:**

**What are the main findings?**

- Across 44 quantitative studies, supportive organizational responses, just and non-punitive cultures, learning-oriented safety climates, and accessible structured support were generally associated with less second-victim distress, more favorable recovery, and fewer negative professional consequences.
- The evidence base was overwhelmingly clinician-centered: 40 of 44 studies assessed distress/recovery and 21 assessed professional consequences, whereas only five evaluated other safety-related outcomes, one evaluated disclosure-related behavior, and none prospectively evaluated incident/near-miss reporting or speaking up.

**What are the implications of the main findings?**

- The available evidence supports consideration of timely, confidential, tiered post-incident support within just-culture and learning-oriented incident-response systems, while the effectiveness of specific organizational models requires prospective and controlled evaluation.
- Future evaluations should use prospective or controlled designs and separately measure clinician recovery, verified workforce outcomes, directly measured safety behaviors, and patient outcomes.

**Abstract:**

**Background/Objectives:** Patient safety incidents can adversely affect healthcare professionals, but relationships between leadership and organizational responses, clinician recovery, professional consequences, and safety-related outcomes remain incompletely synthesized. This review examined contemporary quantitative evidence on these relationships and the extent to which directly measured safety behaviors and patient-safety outcomes have been evaluated. **Methods:** This PROSPERO-registered systematic review (CRD420261453278) followed PRISMA 2020 and PRISMA-S. PubMed and Scopus were searched as the primary bibliographic databases, supplemented by Europe PMC and OpenAlex and by forward citation searching in Scopus and the Web of Science Core Collection, for English-language studies published from January 2021 through July 2026. Two reviewers independently screened records and extracted data; design-specific JBI appraisal judgments were agreed by two reviewers. Heterogeneity precluded meta-analysis, and findings were synthesized narratively using SWiM-informed structured reporting. **Results:** Of 6838 records, 44 studies were included. Distress/recovery was assessed in 40 studies (90.9%), professional consequences in 21 (47.7%), other safety-related outcomes or indicators in five (11.4%), and disclosure-related behavior in one (2.3%). No study prospectively evaluated incident or near-miss reporting or speaking up as a principal exposure-linked outcome. Supportive organizational responses, just and non-punitive cultures, learning-oriented safety climates, and structured support were generally associated with less distress, more favorable recovery, and fewer negative professional outcomes. Structured-support evaluations suggested feasibility, acceptability, and favorable short-term signals but did not establish intervention effectiveness. **Conclusions:** Contemporary evidence is substantially stronger for clinician recovery and professional functioning than for safety behavior or patient outcomes. Predominantly cross-sectional, self-reported, and uncontrolled evidence precludes causal conclusions. Direct evaluation of reporting, disclosure, speaking up, verified workforce outcomes, and patient-safety effects remains a major research priority.

## 1. Introduction

Patient safety incidents remain a major challenge for healthcare systems worldwide. Although the immediate and most serious consequences are experienced by patients and their families, such incidents can also affect the healthcare professionals involved and the organizations in which they occur. The World Health Organization’s Global Patient Safety Action Plan 2021–2030 identifies leadership commitment, a learning-oriented safety culture, workforce protection, reporting systems, and organizational learning as central requirements for reducing avoidable harm. These priorities indicate that the response to a patient safety incident should not be understood solely as an individual matter, but as an organizational process with potential implications for staff well-being, professional practice, and subsequent safety performance [1].

The term “second victim” was introduced to describe physicians who are negatively affected after involvement in a medical error [2]. A subsequent evidence- and consensus-based definition broadened the concept to include any healthcare worker directly or indirectly involved in an unanticipated adverse patient event, an unintentional healthcare error, or patient injury who is negatively affected by that involvement [3]. The phenomenon may encompass guilt, shame, anxiety, intrusive recollections, sleep disturbance, loss of professional confidence, social withdrawal, impaired work functioning, and concerns about continuing clinical practice [4,5]. Recent studies involving nurses, physicians, midwives, respiratory therapists, and other healthcare professionals have documented substantial variation in the nature and severity of second-victim experiences across professional groups, clinical settings, incident types, and career stages [6,7,8,9,10,11,12,13,14]. Positive post-incident outcomes have also been reported. In addition to distress and professional disruption, some affected healthcare professionals report post-traumatic growth [15,16,17].

The term “second victim” remains widely used in the literature, although its terminology has been debated; in this review, it is retained for consistency with the established empirical literature and search terminology, while “affected healthcare professionals” is used where appropriate.

The organizational environment has been associated with healthcare professionals’ post-incident experiences, although the available evidence is predominantly cross-sectional and does not establish causal or temporal relationships. Relevant factors include the responses of supervisors and senior leaders, perceived institutional support, non-punitive management of error, just culture, psychological safety, communication openness, peer support, structured debriefing, interprofessional collaboration, and access to professional referral pathways. Across 396 healthcare work settings, perceptions of stronger institutional support for colleagues affected by unanticipated clinical events were associated with more favorable safety-culture and workforce well-being assessments [18]. In a Chilean cross-sectional study, lower perceived support quality was associated with more severe psychological and occupational consequences following adverse events [19]. Lower organizational support was also associated with more severe second-victim symptom profiles in latent-profile analyses involving Korean and Italian healthcare professionals [20,21].

Patient safety culture represents a broader organizational context within which post-incident experiences develop. Non-punitive responses to error, communication openness, organizational learning, and teamwork have been associated with second-victim distress and professional outcomes [22]. Patient safety culture has also been associated with perceived support and distress and has shown indirect relationships with negative work outcomes through second-victim experiences [23]. Similarly, perceived just culture was associated with lower second-victim distress and lower demand for support in a path model involving nurses who had experienced patient safety incidents [24]. Other quantitative studies have examined colleague, supervisor, and institutional support as potential mediators or correlates of work-related outcomes, resilience, distress, recovery, and unmet support needs [25,26,27,28,29].

Professional consequences constitute a related but analytically distinct outcome domain. Second-victim experiences have been associated with turnover intention, intention to leave patient care or the employing organization, absenteeism, job insecurity, and impaired professional functioning; reduced professional self-efficacy has been examined as a distinct second-victim outcome [6,11,12,27,30,31,32,33,34,35]. Organizational support has been examined both as a direct correlate and as a potential mediator of these relationships. In a Malaysian multicenter study, colleague, supervisor, and institutional support mediated relationships between second-victim distress or professional self-efficacy and negative work-related outcomes or resilience [25]. In a subsequent mediation analysis, perceived organizational support partially mediated the associations between second-victim distress and turnover intention and absenteeism [27]. Patient safety culture and second-victim support have likewise shown indirect associations with negative work outcomes through distress-related pathways [23,24]. Conversely, resilience, professional identity, social support, interprofessional collaboration, and post-traumatic growth may represent protective or recovery-related pathways, although their direction and temporal ordering generally cannot be established from cross-sectional evidence [12,15,16,17,25,27,33,34].

Healthcare organizations have increasingly introduced structured post-incident interventions, including peer-support programs, departmental buddy systems, educational seminars, tiered support models, facilitated debriefing, and organizational-learning processes. Quantitative program evaluations have examined changes in awareness of second-victim resources, perceived availability or adequacy of support, coping-related knowledge, program utilization, safety-culture perceptions, and immediate distress following peer-support encounters [36,37,38,39,40,41,42,43,44]. Some evaluations reported favorable changes in perceived support or short-term distress, but the studies differed substantially in design, sample selection, implementation context, comparator conditions, follow-up periods, and outcome measurement.

These findings point to distinct post-incident outcome pathways. Leadership-related and organizational factors may be associated with second-victim distress and recovery, professional consequences, and safety-related outcomes. These domains should be synthesized separately because improvements in staff support, well-being, recovery, or program utilization cannot be assumed to indicate improved safety behavior or reduced patient harm unless these outcomes are measured directly.

Compared with distress, perceived support, and workforce outcomes, direct quantitative assessment of post-incident changes in clinical practice appears less developed. A structural-equation-modeling study involving clinical nurses examined constructive changes in nursing practice, classified in this review within the safety-related outcome domain, and defensive changes, classified within the professional-consequences domain, and identified pathways involving just culture, second-victim experiences, and coping strategies [45]. Open disclosure has also been investigated in the broader second-victim literature [46]. Nevertheless, directly measured safety behaviors and patient-safety indicators have been examined much less consistently than psychological distress, support needs, and workforce outcomes. These safety-related domains therefore require separate outcome-specific synthesis.

Previous evidence syntheses have examined complementary aspects of the second-victim phenomenon. A recent systematic review evaluated interventions intended to support affected healthcare professionals and identified considerable heterogeneity in intervention content, outcome measurement, study design, and certainty of evidence [47]. Another systematic review examined the support healthcare professionals want, receive, and perceive as effective following patient safety incidents and identified persistent misalignment between staff needs and available organizational responses [48]. A scoping review mapped the validation, application, and limitations of the Second Victim Experience and Support Tool across healthcare settings and professional groups [49]. A qualitative systematic review subsequently examined the reciprocal relationships between individual experiences and organizational responses [50]. In addition, a scoping-review protocol has been published to map leadership factors affecting healthcare professionals as second victims in hospital settings [51].

However, to the authors’ knowledge, the completed reviews have not provided an outcome-specific synthesis restricted to quantitative primary studies examining associations between predefined leadership and organizational exposures and measurable post-incident outcomes. In particular, they have not separately synthesized safety-related reporting and disclosure behaviors, other patient-safety outcomes, second-victim distress and recovery, and professional consequences, nor systematically examined direct, indirect, and mediated pathways across these domains.

The 1 January 2021 start date was prespecified to focus the systematic synthesis on the contemporary quantitative evidence base rather than reconstruct the full historical development of the second-victim literature. This evidence window also coincides with the current global patient-safety policy era represented by the WHO Global Patient Safety Action Plan 2021–2030 [1]. Earlier foundational peer-support evaluations, conceptual work, and instrument-development studies remain important to the development of the field and were used for contextualization where relevant, but were not part of the systematic synthesis unless they met the prespecified publication-period criterion. The present review should therefore be interpreted as a contemporary evidence synthesis rather than a complete historical review or a formal update of a single previous systematic review. The review question was: Among healthcare professionals affected by patient safety incidents, how are leadership-related and organizational factors associated with prespecified post-incident outcomes across second-victim distress and recovery, professional consequences, reporting and disclosure behaviors, and other safety-related outcomes and patient-safety indicators?

The objective of this systematic review was to identify, critically appraise, and synthesize quantitative evidence concerning associations between leadership-related and organizational factors following patient safety incidents across four prespecified outcome domains: (1) safety-related reporting and disclosure behaviors, including incident and near-miss reporting, speaking up, and open disclosure; (2) other safety-related outcomes and patient-safety indicators, including safety participation, safety performance, organizational learning, repeated adverse events, and validated safety indicators or composite measures, where reported; (3) second-victim distress, coping, recovery, post-traumatic growth, professional confidence, and self-efficacy; and (4) professional consequences, including turnover intention, intention to leave clinical practice or the organization, absenteeism, burnout, job satisfaction, organizational commitment, defensive clinical practice, and reduced professional functioning. Direct associations and statistically modeled indirect or mediated pathways were examined where reported.

### Clinical and Organizational Significance

Understanding how leadership and organizational responses are associated with post-incident outcomes is relevant to healthcare executives, clinical and nursing managers, patient-safety professionals, occupational-health services, and teams responsible for second-victim support programs. An outcome-specific synthesis can help organizations distinguish interventions intended primarily to reduce healthcare professionals’ distress from organizational practices associated with reporting, disclosure, speaking up, safety participation, or other measurable safety-related outcomes. This distinction is important because evidence of improved perceived support, well-being, recovery, or program utilization should not be interpreted as evidence of improved patient safety unless corresponding safety behaviors or indicators have been directly assessed. By examining direct associations alongside statistically modeled indirect and mediated pathways, this review was designed to clarify the extent and limitations of the available quantitative evidence and to identify priorities for leadership practice, organizational policy, and future research.

## 2. Materials and Methods

### 2.1. Study Design, Registration, and Reporting Framework

This systematic review examined associations between leadership-related and organizational responses following patient safety incidents and prespecified outcomes among affected healthcare professionals. The scope and methodological approach were defined a priori using a Population, Exposure, Comparator, Outcomes, and Study design (PECOS) framework. The review distinguished safety-related reporting and disclosure behaviors and other patient-safety outcomes from second-victim distress and recovery outcomes and from professional consequences.

The review was registered in the International Prospective Register of Systematic Reviews (PROSPERO; registration number CRD420261453278) on 17 July 2026. The review question, PECOS eligibility criteria, publication period, exposure categories, outcome domains, eligible study designs, and planned outcome-specific synthesis framework were prespecified in the registration.

Following PROSPERO registration on 17 July 2026 and before completion of the final searches on 30 July 2026, the planned information sources were amended. The original registration listed CINAHL, Embase, MEDLINE, APA PsycINFO, PubMed, and Scopus. During implementation of the review, reproducible institutional access and bibliographic export were not available for CINAHL, Embase, or APA PsycINFO through the institutional access routes available to the review team. PubMed, including MEDLINE-indexed records, and Scopus were therefore retained as the primary bibliographic databases; Europe PMC and OpenAlex were used as supplementary open-index sources; and the Web of Science Core Collection was used for forward citation searching rather than as a primary bibliographic database. The search-source amendment did not alter the review question, core PECOS framework, publication period, exposure categories, prespecified outcome domains, eligible study designs, or outcome-specific synthesis framework. The PROSPERO record was updated during peer-review revision to document the completed search workflow and review status, remove planned backward citation searching that was not undertaken, and clarify the publication-type eligibility wording to reflect the completed review.

No standalone protocol article was published. The Preferred Reporting Items for Systematic Review and Meta-Analysis Protocols (PRISMA-P) 2015 statement informed the prospective specification and documentation of the review methods [52]. Reporting of the completed review followed the Preferred Reporting Items for Systematic Reviews and Meta-Analyses (PRISMA) 2020 statement [53]. Methodological appraisal was guided by the JBI Manual for Evidence Synthesis, using the critical appraisal instrument appropriate to the design of each included study [54]. The search methods were reported in accordance with the PRISMA extension for reporting literature searches in systematic reviews (PRISMA-S) [55]. The full source-specific search strategies are provided in Supplementary File S1; the completed PRISMA 2020 checklist is provided in Supplementary File S2; detailed study characteristics and PECOS classifications are provided in Supplementary Table S1; the complete item-level JBI critical-appraisal judgments are provided in Supplementary Table S2; and the reports excluded after full-text assessment, with their primary reasons for exclusion, are provided in Supplementary Table S3.

### 2.2. Information Sources and Search Strategy

PubMed, including MEDLINE-indexed records, and Scopus were searched as the primary bibliographic databases because they provide broad biomedical and interdisciplinary coverage relevant to healthcare professionals, patient safety, organizational factors, and health-services research. Europe PMC and OpenAlex were used as supplementary open-index sources to broaden retrieval beyond the primary databases, rather than as equivalent substitutes for discipline-specific databases. Forward citation searching of included studies and key reviews was additionally conducted in Scopus and the Web of Science Core Collection. CINAHL, Embase, and APA PsycINFO were not searched because reproducible institutional access and bibliographic export were not available through the institutional access routes available to the review team. The 2021 start date was prespecified to define a contemporary evidence window; the review was therefore intended to characterize current evidence rather than provide a complete historical synthesis of the second-victim literature.

The search strategy combined controlled vocabulary, where available, with free-text terms relating to four principal concepts: patient safety incidents; the second-victim phenomenon; healthcare professionals; and leadership-related or organizational responses. Search terms included variations of “second victim”, “second-victim syndrome”, “second-victim phenomenon”, “patient safety incident”, “adverse event”, “medical error”, “near miss”, “healthcare professional”, “nurse”, “physician”, “organizational support”, “institutional support”, “leadership”, “managerial response”, “just culture”, “patient safety culture”, “psychological safety”, “communication openness”, “peer support”, “debriefing”, and “organizational learning”. British and American spelling variants, singular and plural forms, phrase searching, truncation, and database-specific field codes were applied as appropriate.

Two complementary search approaches were used. The first applied a high-sensitivity second-victim concept without requiring an organizational or outcome term. The second combined patient-safety-incident terminology with healthcare-professional and leadership-related or organizational concepts to identify potentially eligible studies that did not explicitly use the term “second victim”. No outcome or study-design filter was applied because eligible outcomes and methodological designs were described inconsistently across the literature. English-language limits were applied in PubMed, Scopus, and Europe PMC. OpenAlex was searched without a language filter; the English-language eligibility criterion was applied during screening.

The PubMed strategy was developed as the reference strategy and was adapted to the controlled vocabulary, indexing structure, field codes, proximity operators, and syntax of each database. The complete source-specific strategies, including the platform, exact search string, date of execution, applied limits, and number of records retrieved, are provided in Supplementary File S1.

Forward citation searching of the included studies and key reviews was conducted in Scopus and the Web of Science Core Collection. Records identified through citation searching were imported into the structured master database and underwent the same deduplication, screening, and report-level eligibility-assessment procedures as records identified through the electronic database searches.

### 2.3. Eligibility Criteria

Eligibility criteria were operationalized before formal screening using the Population, Exposure, Comparator, Outcomes, and Study design (PECOS) framework presented in Table 1. Eligible studies involved practicing healthcare professionals who had been directly or indirectly affected by a patient safety incident and examined the relationship between at least one leadership-related or organizational factor and at least one prespecified post-incident outcome. Eligibility required the assessment of at least one of the four prespecified outcome domains; evaluation of the reporting and disclosure domain was not mandatory for study inclusion. A formal comparator was not required for observational studies, provided that an association between an eligible organizational exposure and an eligible outcome was quantitatively assessed.

Peer-reviewed primary studies published from 1 January 2021 through the final source-specific search date in July 2026 were eligible. Only articles published in English were eligible for inclusion. Studies conducted in any healthcare profession, clinical specialty, healthcare setting, or country were considered, provided that the population, exposure, and incident-related eligibility criteria were satisfied. Eligible designs comprised analytical cross-sectional, prospective or retrospective cohort, longitudinal, case–control, quasi-experimental, controlled or uncontrolled intervention, and randomized controlled studies, together with the quantitative components of mixed-methods studies.

Studies were excluded when they involved exclusively undergraduate or pre-licensure students; examined occupational stress, burnout, workplace violence, or other traumatic experiences without an identifiable patient safety incident; or did not assess an eligible leadership-related or organizational factor. Systematic, scoping, narrative, umbrella, and integrative reviews; qualitative-only studies; study protocols; editorials; letters; commentaries; conference abstracts, dissertations, and theses were excluded. Instrument-development or validation studies were excluded unless they also evaluated an eligible exposure–outcome relationship. Descriptive studies reporting only the prevalence, frequency, or severity of second-victim experiences, without examining an eligible organizational or leadership factor, were also excluded.

The English full-text criterion was applied during the full-text eligibility assessment. Earlier foundational studies and methodological or instrument-development publications could be used to contextualize the review and interpret measurement approaches, but they were not included in the systematic synthesis unless they met all prespecified eligibility criteria.

### 2.4. Study Selection, Data Extraction, and Risk-of-Bias Assessment

All records identified through the electronic database searches and supplementary study-identification methods were imported into a structured master database. Duplicate records were identified by comparing Digital Object Identifiers (DOIs), PubMed identifiers (PMIDs), exact and normalized titles, author names, publication years, journal titles, and page or article numbers. Records with uncertain duplicate status were examined manually before removal.

Two reviewers independently screened each title and abstract against the prespecified eligibility criteria. Records were retained for report-level eligibility assessment whenever the available information was insufficient to support exclusion. Reports retained after title and abstract screening were independently assessed in full text by two reviewers against the complete PECOS eligibility criteria. Disagreements at either stage were resolved through discussion and consensus; when consensus could not be reached, a third reviewer was consulted. One primary reason was recorded for each report excluded after full-text assessment, and reports that could not be retrieved were recorded separately.

Reports arising from the same underlying study or participant sample were identified by comparing authorship, country, healthcare setting, recruitment period, sample size and participant characteristics, measurement instruments, intervention or exposure descriptions, funding information, and study procedures. Multiple reports relating to the same underlying study were linked and counted as one study in study-level totals. Unique eligible outcome data reported across associated publications were retained, whereas overlapping participant data and findings were not counted more than once.

Data were independently extracted by two reviewers using a standardized extraction form developed for this review. The form captured bibliographic information; country and healthcare setting; study design and recruitment period; professional group; sample size and participant characteristics; definition and type of patient safety incident; leadership-related and organizational exposures; comparator characteristics, where applicable; intervention components and implementation features; outcome domains and measurement instruments; assessment time points; statistical methods; adjusted and unadjusted effect estimates; confidence intervals and *p*-values, where reported; variables included in multivariable, path, or mediation models; principal findings; funding sources; and reported conflicts of interest. The completed extraction records were compared, and discrepancies were resolved through re-examination of the source report and consensus, with consultation with a third reviewer when required.

For mixed-methods studies, only data relating to the eligible quantitative component were extracted. Information reported across linked publications was compared to obtain the most complete study-level record and to prevent duplicate counting. Study authors were not contacted to obtain or clarify missing information. Information that was absent or insufficiently described was recorded as “not reported” or “unclear”, as appropriate, and unreported numerical values were not imputed.

Design-specific critical appraisal was conducted using the applicable JBI instruments [54]. The instrument appropriate to the underlying quantitative design of each study was applied. Each applicable appraisal item was recorded as “yes”, “no”, “unclear”, or “not applicable”, together with a study-specific justification. The item-level judgments were reviewed and agreed upon by two reviewers, with discrepancies resolved through discussion and consensus. No composite numerical score or arbitrary quality threshold was used. Studies were not excluded solely on the basis of their risk-of-bias assessment. Item-level judgments were considered when interpreting the credibility, consistency, and limitations of the evidence within each outcome domain. Complete study-level and item-level judgments are reported in the Supplementary Materials.

### 2.5. Outcomes

The outcome framework was prespecified in the PROSPERO record and organized into four distinct domains. The primary outcome domain comprised safety-related reporting and disclosure behaviors, including actual reporting of adverse events or near misses, willingness or intention to report patient safety incidents, speaking up about patient-safety concerns, and open disclosure to patients or their families.

The first secondary domain comprised other safety-related outcomes and patient-safety indicators, including safety participation, safety performance, engagement in organizational learning following incidents, repeated healthcare errors or adverse events, and validated patient-safety indicators or composite measures. The second secondary domain comprised second-victim outcomes, including psychological, physical, and professional distress; symptom severity and duration; coping and recovery; post-traumatic growth; professional confidence and self-efficacy; and perceived adequacy of post-incident support. The third secondary domain comprised professional consequences, including turnover intention, intention to leave clinical practice or the employing organization, absenteeism, burnout, job satisfaction, organizational commitment, defensive clinical practice, and reduced professional functioning. The four outcome domains were synthesized separately and were not combined into a single overall outcome. A study was eligible when it evaluated at least one of these four domains; measurement of the primary reporting and disclosure domain was not required.

For each included study, outcomes were recorded according to the definitions and measurement instruments used by the study authors. The instrument or measure used, its validation status, score range and direction, assessment time point, and reported effect measure were extracted where available. When a study reported several eligible measures or assessment time points, each was retained and classified within the corresponding outcome domain. Adjusted estimates were prioritized for comparative synthesis when both adjusted and unadjusted estimates were available; unadjusted estimates were retained when no adjusted analysis was reported. Direct, indirect, and total effects from path or mediation models were recorded separately.

Patient safety culture, psychological safety, communication openness, and organizational-learning climate measures were generally treated as leadership-related or organizational exposures or contextual variables. Changes in these organizational constructs were classified as outcomes only when a study explicitly evaluated them following a leadership-related or organizational intervention. In contrast, healthcare professionals’ engagement in post-incident organizational learning activities was classified within the other safety-related outcomes and patient-safety indicators domain. Perceived organizational support, support adequacy, and resource availability were treated as exposures when analyzed as predictors or contextual organizational factors. They were classified as outcomes only when a study explicitly evaluated change in these constructs following implementation of a leadership-related or organizational intervention. Program awareness, reach, uptake, utilization, satisfaction, and perceived usefulness did not independently satisfy the outcome eligibility criterion. When reported by studies that met the eligibility criteria through at least one of the four prespecified outcome domains, these variables were extracted and summarized separately as implementation outcomes. They were not interpreted as evidence of improved reporting, disclosure, safety behavior, safety performance, or patient outcomes unless those outcomes had been measured directly.

### 2.6. Statistical Analysis and Synthesis

The characteristics and findings of the included studies were summarized using descriptive statistics and structured evidence tables. Categorical variables, including country, professional group, healthcare setting, study design, exposure category, and outcome domain, were summarized using frequencies and percentages where appropriate. Leadership-related and organizational exposure domains and outcome domains were non-mutually exclusive; therefore, individual studies could contribute to more than one category, and the corresponding frequencies and percentages were not expected to sum to the total number of studies or to 100%. Continuous study characteristics were reported using the summary measures provided by the original studies and were not recalculated when the necessary participant-level data were unavailable.

The evidence was synthesized separately across the four prespecified outcome domains: (1) safety-related reporting and disclosure behaviors; (2) other safety-related outcomes and patient-safety indicators; (3) second-victim distress and recovery outcomes; and (4) professional consequences. Within each outcome domain, studies were further grouped according to five non-mutually exclusive leadership-related or organizational exposure domains: (1) leadership and supervisory responses; (2) organizational and institutional support; (3) just culture and non-punitive response; (4) patient safety culture, psychological safety, communication openness, and organizational learning; and (5) structured second-victim support, peer support, debriefing, and referral systems.

The synthesis retained the effect measures reported by the individual studies, including correlation coefficients, regression coefficients, odds ratios, risk estimates, mean differences, standardized coefficients, and direct, indirect, and total effects derived from path or mediation analyses. Adjusted effect estimates were prioritized when both adjusted and unadjusted results were available. Unadjusted estimates were retained when no adjusted analysis was reported. Effect estimates were interpreted together with their confidence intervals, *p*-values, model specifications, sample characteristics, and relevant risk-of-bias judgments.

Effect estimates were not converted to a common metric or mathematically transformed. Numerical effect estimates were retained exactly as reported by the original studies. Where instruments used opposite scoring directions, the direction of association was described in standardized verbal terms for the narrative synthesis, without mathematical transformation of the original estimate.

Meta-analysis was not performed because substantial clinical, conceptual, methodological, and measurement heterogeneity was identified across the included studies. Sources of heterogeneity included differences in professional groups and healthcare settings, definitions of patient safety incidents, leadership and organizational exposures, outcome constructs, measurement instruments, assessment time points, study designs, statistical models, and reported effect measures. The available studies therefore could not be combined into sufficiently homogeneous quantitative groups without risking clinically or conceptually misleading pooled estimates. Because no quantitative pooling was undertaken, no formal subgroup analysis, meta-regression, or sensitivity analysis was performed.

A structured narrative synthesis was consequently undertaken, with reporting informed by the Synthesis Without Meta-analysis (SWiM) guideline where applicable [56]. For each exposure–outcome grouping, the synthesis examined the direction, magnitude, precision, and consistency of reported associations. Statistically significant and non-significant findings were both retained, and conclusions were not based solely on the number of statistically significant results. Where findings differed across studies, possible explanations were examined in relation to professional group, healthcare setting, study design, exposure definition, measurement approach, analytical adjustment, and risk-of-bias concerns. To make the synthesis more transparent, a SWiM-informed structured summary display was used to distinguish evidence volume and overall narrative pattern from the methodological constraints affecting interpretation; no vote counting based on statistical significance was undertaken.

Direct associations were distinguished from indirect or mediated pathways. Findings from mediation, path-analysis, and structural-equation models were synthesized separately from conventional bivariate and multivariable associations because these approaches represented different causal assumptions and effect structures. Multiple reports based on the same underlying study population were linked and were not counted as independent evidence when summarizing the direction or consistency of findings.

Implementation outcomes, including program awareness, reach, uptake, utilization, satisfaction, and perceived usefulness, were summarized separately from safety-related, second-victim, and professional outcomes. Improvements in implementation outcomes or perceived organizational support were not interpreted as evidence of improved incident reporting, disclosure, safety performance, or patient outcomes unless these outcomes had been directly measured. Formal statistical or graphical assessment of risk of bias due to missing results was not undertaken because the studies within the exposure–outcome groupings were not sufficiently comparable in design, measures, and reported effect estimates to support a meaningful assessment.

Formal certainty-of-evidence grading was not undertaken because the review included heterogeneous observational, mediation/path-model, quasi-experimental, and program-evaluation evidence across conceptually distinct exposure–outcome groupings. Narrative confidence in the interpretation of each outcome domain was therefore assessed explicitly. Greater confidence was placed in findings supported by multiple studies showing directionally consistent associations, more complete adjustment for relevant confounding, greater precision, direct applicability to the review question, and fewer material concerns in the design-specific JBI appraisal. Confidence was reduced when evidence was sparse, based predominantly on cross-sectional self-report, affected by important JBI concerns, inconsistent or imprecise, or derived from uncontrolled or non-contemporaneous intervention evaluations. These considerations informed the strength of the wording used in the Results, Discussion, and Conclusions; volume or directional consistency of evidence was not treated as equivalent to causal certainty.

## 3. Results

### 3.1. Study Selection

A total of 6838 records were identified through electronic searches across four sources: PubMed (*n* = 2497), Scopus (*n* = 2524), Europe PMC (*n* = 1538), and OpenAlex (*n* = 279). Forward citation searching of the included studies and key reviews in Scopus and the Web of Science Core Collection did not identify any additional studies meeting the eligibility criteria; the total number of citation-searching records was not retained separately. Before screening, 3137 duplicate records and a further 193 records that fell outside the prespecified publication period or did not provide a usable publication year were removed. Consequently, 3508 unique records underwent title and abstract screening.

During title and abstract screening, 3443 records were excluded because they did not meet the prespecified PECOS eligibility criteria. Sixty-five reports were sought for retrieval; all 65 were retrieved and assessed for full-text eligibility. Twenty-one reports were excluded after full-text assessment: no eligible leadership-related or organizational exposure–outcome analysis (*n* = 14), wrong population or no separable subgroup of healthcare professionals affected by a patient safety incident (*n* = 4), and implementation-only evaluation without an eligible prespecified post-incident outcome (*n* = 3). The 21 reports excluded after full-text assessment and their primary reasons for exclusion are listed in Supplementary Table S3.

**Figure 1:**
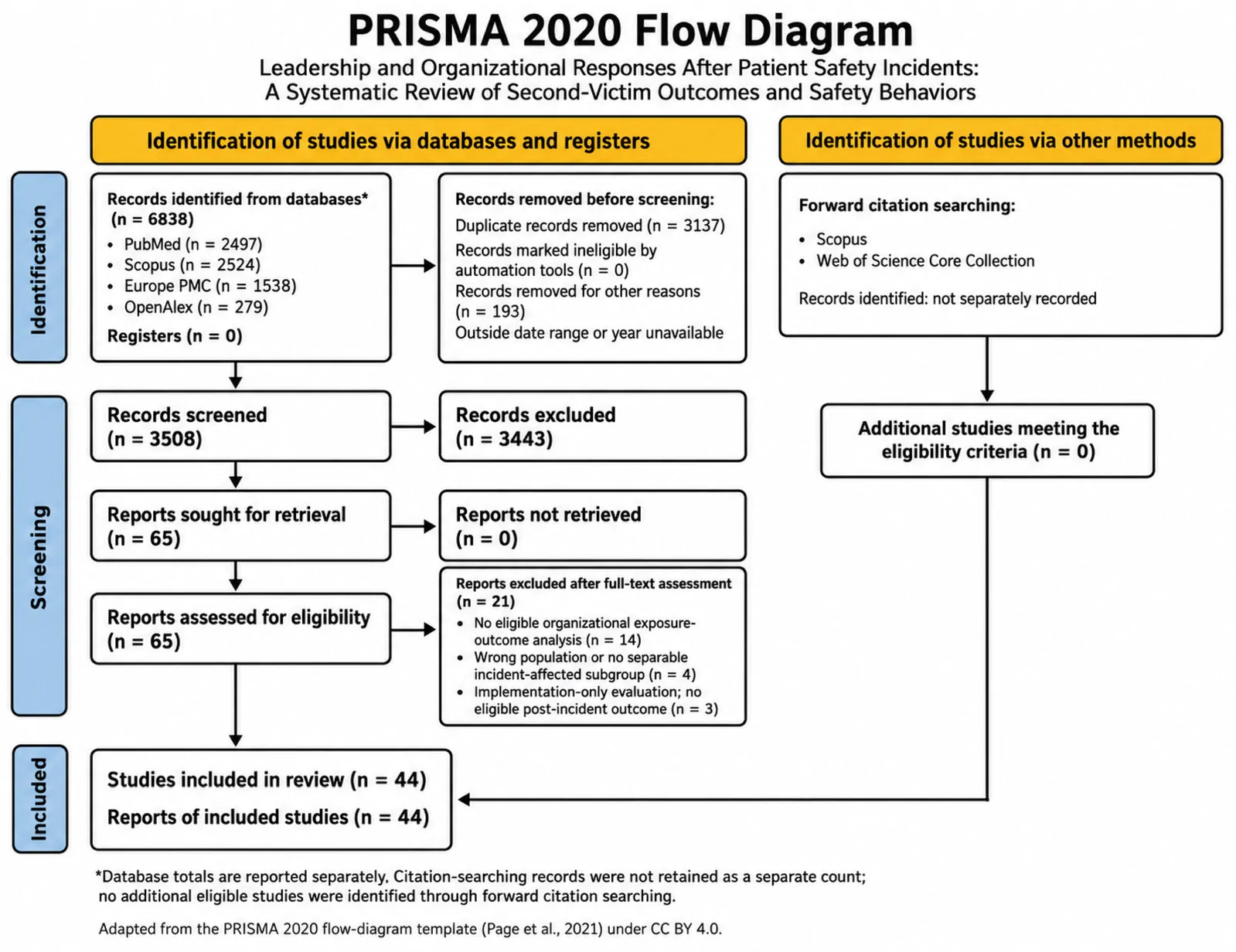
PRISMA 2020 flow diagram of study identification, screening, full-text eligibility assessment, and inclusion in the systematic review. Citation-searching records were not retained as a separate count; no additional eligible studies were identified through forward citation searching. Adapted from Page et al. [53] under CC BY 4.0.

Forty-four reports, representing 44 distinct studies, met all eligibility criteria and were included in the review and structured narrative synthesis. No multiple included reports arising from the same underlying study population were identified. The study-selection process is presented in Figure 1.

### 3.2. Characteristics and PECOS-Aligned Evidence Domains of the Included Studies

**Figure 2:**
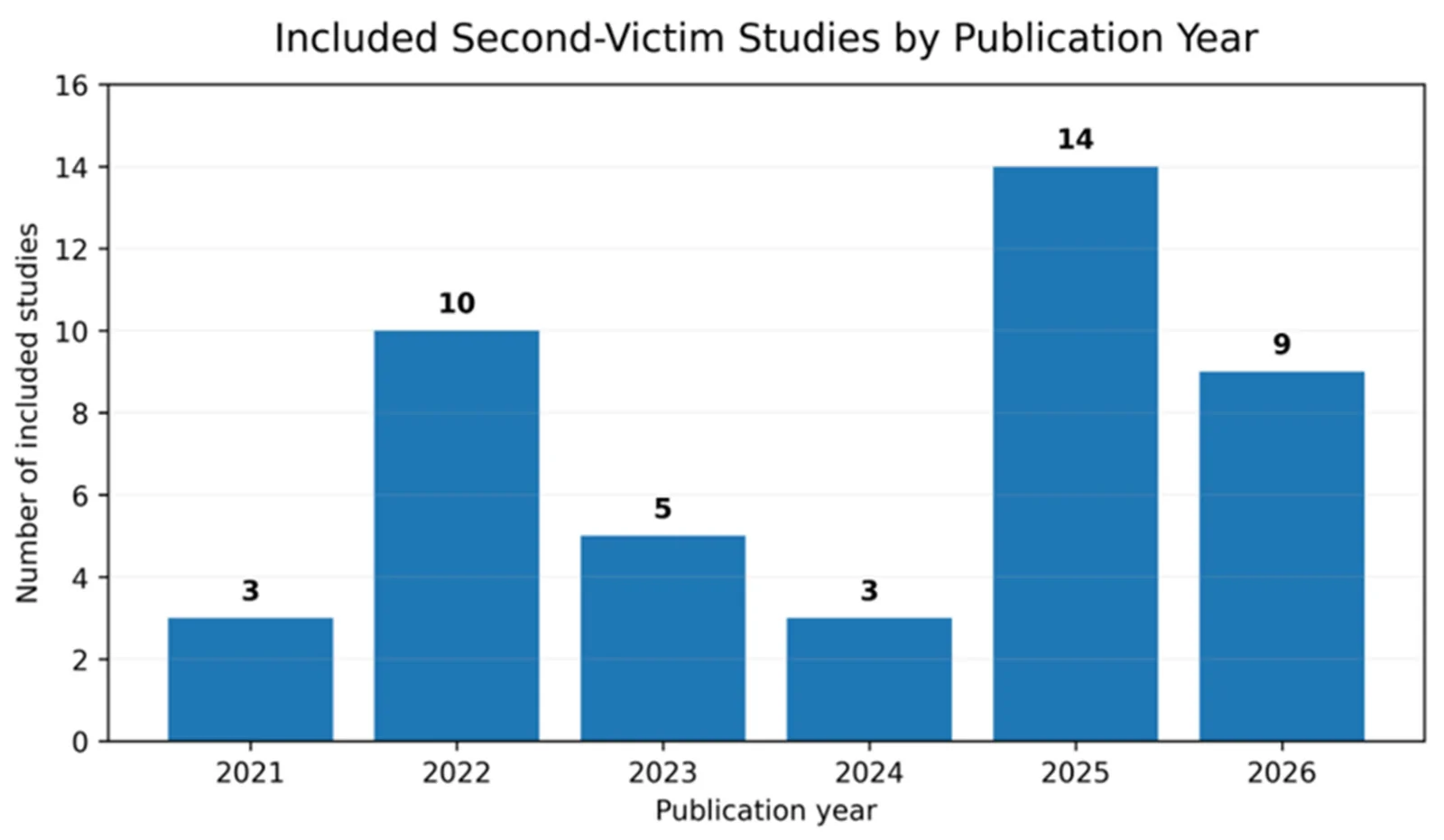
Publication-year distribution of the 44 included studies examining leadership-related and organizational responses following patient safety incidents. The 2026 count reflects partial-year evidence retrieval through 30 July 2026 and does not represent a complete calendar year.

The final synthesis comprised 44 reports representing 44 distinct studies published between 2021 and 2026 [15,16,17,19,20,21,22,23,24,25,27,28,30,31,33,36,37,39,42,45,57,58,59,60,61,62,63,64,65,66,67,68,69,70,71,72,73,74,75,76,77,78,79,80]. The annual distribution was three studies in 2021, 10 in 2022, five in 2023, three in 2024, 14 in 2025, and nine in 2026. Twenty-three studies (52.3%) were published in 2025–2026. The 2026 count reflects evidence available through the final search date and does not represent a complete calendar year (Figure 2).

The evidence base was geographically broad but concentrated in hospital-based settings. Studies were conducted across Asia, Europe, North America, Latin America, and the Middle East, with additional multinational samples. Most were undertaken in tertiary or general hospitals, intensive-care and emergency departments, perioperative and anesthesiology services, pediatric settings, and multidisciplinary clinical departments. Twenty-nine studies (65.9%) focused predominantly on nurses, six (13.6%) examined physicians, anesthesiology professionals, or neurointerventionalists, and nine (20.5%) included multidisciplinary healthcare professionals or mixed clinical staff. The evidence was therefore strongly nursing- and hospital-centered, with comparatively limited representation of primary-care, community, and non-hospital practice.

Thirty-two studies (72.7%) used observational survey-based designs, 11 (25.0%) evaluated an organizational intervention, support program, or incident-response initiative, and one (2.3%) was a mixed-methods study with an eligible quantitative component. Twelve studies applied multivariable path analysis, mediation analysis, structural equation modeling, or latent-profile/latent-class methods [20,21,23,24,27,31,45,60,62,72,73,75]. The intervention studies evaluated structured peer-support systems, restorative just-culture approaches, mindfulness-based support, external support services, and department- or system-level post-incident pathways [36,37,39,42,63,64,65,66,67,69,70]. Controlled evaluations were uncommon, and the evidence base remained dominated by cross-sectional associations and uncontrolled pre–post or quality-improvement evaluations.

The leadership-related and organizational exposures were classified into five non-mutually exclusive domains. Organizational and institutional support was the most frequent domain (26/44, 59.1%), followed by structured second-victim support, peer support, debriefing, and referral systems (15/44, 34.1%); patient safety culture, psychological safety, communication openness, and organizational learning (14/44, 31.8%); just culture and non-punitive response (8/44, 18.2%); and leadership and supervisory responses (4/44, 9.1%). Because studies could contribute to more than one exposure domain, these percentages were not mutually exclusive.

Outcome coverage was substantially less balanced. Forty studies (90.9%) assessed second-victim distress and recovery outcomes, including distress, coping, resilience, post-traumatic growth, professional self-efficacy, or the perceived adequacy of support. Twenty-one studies (47.7%) examined professional consequences, including turnover intention, intention to leave, absenteeism, burnout, job insecurity, defensive practice, or reduced professional functioning. Five studies (11.4%) evaluated other safety-related outcomes or indicators, including organizational learning, incident-review quality, safety-culture change following an intervention, or practice-related safety behaviors. One included study (2.3%) evaluated disclosure-related behavior in relation to patient safety culture; no study prospectively evaluated incident or near-miss reporting or speaking up as a principal exposure-linked outcome. The PECOS-aligned evidence map in Table 2 therefore demonstrates a clear concentration on clinician-centered recovery and workforce outcomes, along with a major evidence gap in directly measured reporting, disclosure, and safety-behavior outcomes.

Detailed study-level characteristics, numbered references, and PECOS classifications for all 44 included studies are provided in Supplementary Table S1.

### 3.3. Safety-Related Reporting, Disclosure, and Other Patient-Safety Outcomes

Direct evidence for the prespecified reporting and disclosure domain was extremely limited rather than absent. One study (2.3%) assessed disclosure-related behavior in relation to patient safety culture [71]. No study prospectively evaluated actual incident- or near-miss-reporting frequency, willingness or intention to report, or speaking up as a principal exposure-linked outcome. Five studies (11.4%) reported other safety-related outcomes or patient-safety indicators, comprising constructive changes in nursing practice, incident-review quality and recommendation strength, perceived organizational learning after an adverse event, reported adverse-event counts, and patient-safety-culture ratings [45,63,66,69,70]. Because these studies differed in design, intervention content, outcome definition, and analytical approach, their findings were synthesized narratively rather than pooled.

In a cross-sectional survey of Romanian nurses, 12.5% reported having informed a patient about an adverse event. Higher patient-safety-culture scores were associated with a greater likelihood of apologizing to patients or their families (OR = 1.23, 95% CI 1.14–1.33, *p* < 0.001). The source report also described a statistically significant association between safety culture and informing patients, but the stated direction conflicted with the reported odds ratio (OR = 1.10, 95% CI 1.00–1.20); that estimate was therefore not interpreted directionally [71].

In a nationwide cross-sectional study of 461 clinical nurses, partial least-squares structural equation modeling identified pathways linking just culture with post-incident changes in nursing practice [45]. Just culture showed a positive indirect association with constructive practice change through second-victim experience and avoidant coping (B = 0.07, *p* < 0.001), whereas a separate modeled pathway linked lower just-culture conditions with fewer constructive changes (B = −0.12, *p* < 0.001). These modeled associations support a potential organizational pathway from fair, non-punitive incident management to constructive practice adaptation; however, the cross-sectional design does not establish temporal ordering or causality.

A quality-improvement evaluation of a restorative just-culture incident-response framework reported improvements in several review-process indicators [63]. Following implementation, the mean number of recommendations per reviewed incident increased from 1.9 to 3.9 (t(56) = −3.47, *p* = 0.001). Recommendation strength also improved according to both the incident-review teams (chi-square(2) = 7.976, *p* = 0.019) and independent auditors (chi-square(2) = 6.644, *p* = 0.036). In addition, 96.0% of post-implementation recommendations included a plan to verify implementation, compared with 64.8% before implementation (*p* < 0.001). Nevertheless, auditors continued to classify 61.3% of post-implementation recommendations as weak. The evaluation therefore suggests improvement in organizational learning and review outputs, but its uncontrolled before-after design and the absence of a pre-implementation process audit limit attribution of the observed changes to the framework alone.

Two peer-support evaluations reported safety-related organizational perceptions rather than directly observed patient outcomes. In an anesthesiology department, clinicians surveyed after implementation of a skilled peer-support program had higher adjusted odds of agreeing that the organization had learned from the adverse event and had taken steps to prevent recurrence than clinicians surveyed before implementation (adjusted odds ratio 3.9, 95% confidence interval 1.01–15.1) [66]. The wide confidence interval indicates substantial imprecision. In a children’s healthcare system, staff experiencing emotional distress after harm events who reported being offered peer support rated patient safety culture more favorably than those who were not offered support, both overall (mean 3.60 vs. 3.19, *p* < 0.0001) and within their local unit or work area (mean 3.68 vs. 3.34, *p* = 0.001) [70]. These findings indicate favorable associations with perceived learning and safety culture, but neither study used randomized exposure assignment, and both remain susceptible to selection, response, and residual confounding.

A non-contemporaneous case–control evaluation of a multicomponent mindfulness-based second-victim support program for intensive-care nurses reported that adverse-event cases in the intervention group decreased from 50 before implementation to 33 after implementation, corresponding to a 34% reduction [69]. The intervention also incorporated changes to adverse-event reporting procedures and non-punitive management practices. Because the comparison groups were drawn from different calendar years, follow-up was short, and the reported safety result was presented as a within-program event-count reduction without a comparative effect estimate, the finding should be interpreted cautiously and cannot be attributed specifically to the mindfulness component.

Overall, the available safety-related evidence was sparse and methodologically heterogeneous. The findings suggest that learning-oriented incident responses, just-culture practices, and structured support systems may be associated with more constructive post-incident practice, stronger incident-review outputs, more favorable perceptions of organizational learning and safety culture, and fewer reported adverse events. However, disclosure-related behavior was evaluated in only one cross-sectional study, no study prospectively evaluated incident or near-miss reporting or speaking up, and the available evidence does not establish reductions in patient harm. The safety-related findings should therefore be considered preliminary and hypothesis-generating. Study-level results and their principal interpretive constraints are summarized in Table 3.

### 3.4. Second-Victim Distress, Recovery, and Post-Traumatic Growth

Second-victim distress and recovery constituted the dominant outcome domain, addressed in 40 of the 44 included studies (90.9%). Within this subset, organizational and institutional support was examined in 24 studies, structured second-victim support or related post-incident pathways in 14, patient safety culture, psychological safety, communication openness, or organizational learning in 13, just culture or non-punitive responses in seven, and leadership or supervisory responses in four; these exposure categories were not mutually exclusive. Twenty-nine studies used observational or survey-based designs, 10 evaluated an intervention or support program, and one used a mixed-methods design with an eligible quantitative component.

Across the observational evidence, less favorable perceptions of organizational support, lower support quality, or limited access to post-incident resources were generally associated with more severe second-victim symptoms, less favorable symptom profiles, or weaker recovery-related resources [19,20,21,25,28,30,31,57,58,60,61,62,68,72,74,75,78,79,80]. Latent-profile and latent-class studies further indicated substantial heterogeneity among affected professionals in symptom severity, depressive-symptom patterns, and preferred support strategies [20,21,31,72]. Because studies used different versions of the Second Victim Experience and Support Tool and other instruments with direction-specific scoring, associations were interpreted according to the original scale definitions rather than converted to a common metric.

Safety-culture and just-culture studies were generally directionally consistent with lower distress or more favorable recovery-related outcomes, although null findings were also present and the evidence remained predominantly cross-sectional [16,22,23,24,58,62,71,73,75,76]. For example, Eslami et al. [58] found that patient safety culture was associated with perceived support resources but not with distress-related dimensions, illustrating that favorable organizational context did not translate uniformly across all measured outcomes. Path and mediation analyses in other studies suggested possible pathways through coping, psychological capital, professional efficacy, and perceived support, but the concurrent measurement of exposure, mediator, and outcome precluded firm temporal interpretation [23,24,25,27,73,80].

Several studies examined relational and psychological resources that may shape recovery. Organizational support, interprofessional collaboration, psychological empowerment, resilience, social support, cognitive reappraisal, and psychological capital were associated with distress severity, depressive-symptom profiles, or positive adaptation across different settings [15,17,25,27,33,72,80]. These findings support plausible recovery pathways but do not establish mediation as a causal mechanism because most models were cross-sectional.

The intervention evidence included peer-support services, departmental programs, external counseling, mindfulness-based support, restorative incident-response approaches, and other formal pathways [36,37,39,42,63,64,65,66,67,69]. Evaluations generally reported improved awareness or perceived availability of support and selected favorable short-term psychological signals. However, most studies used small samples, non-randomized allocation, unmatched or uncontrolled pre–post designs, or incomplete follow-up. Program reach, exposure, and fidelity were also inconsistently reported. Accordingly, these evaluations are most informative regarding feasibility, acceptability, implementation, and short-term promise rather than established intervention effectiveness.

Positive recovery was less frequently examined than distress. Three studies specifically investigated post-traumatic growth in relation to organizational support, collaboration, safety culture, or social support [15,16,17]. These studies indicate that distress and positive adaptation may coexist and should not be treated as opposite ends of a single continuum.

Overall, the distress/recovery domain contained the largest and most directionally consistent body of evidence in the review. Nevertheless, confidence in causal interpretation remained limited because the evidence was dominated by cross-sectional self-report, heterogeneous measurement, inconsistent adjustment for confounding, and relatively weak longitudinal or controlled intervention designs. Detailed quantitative estimates are retained in the study-level tables.

**Figure 3:**
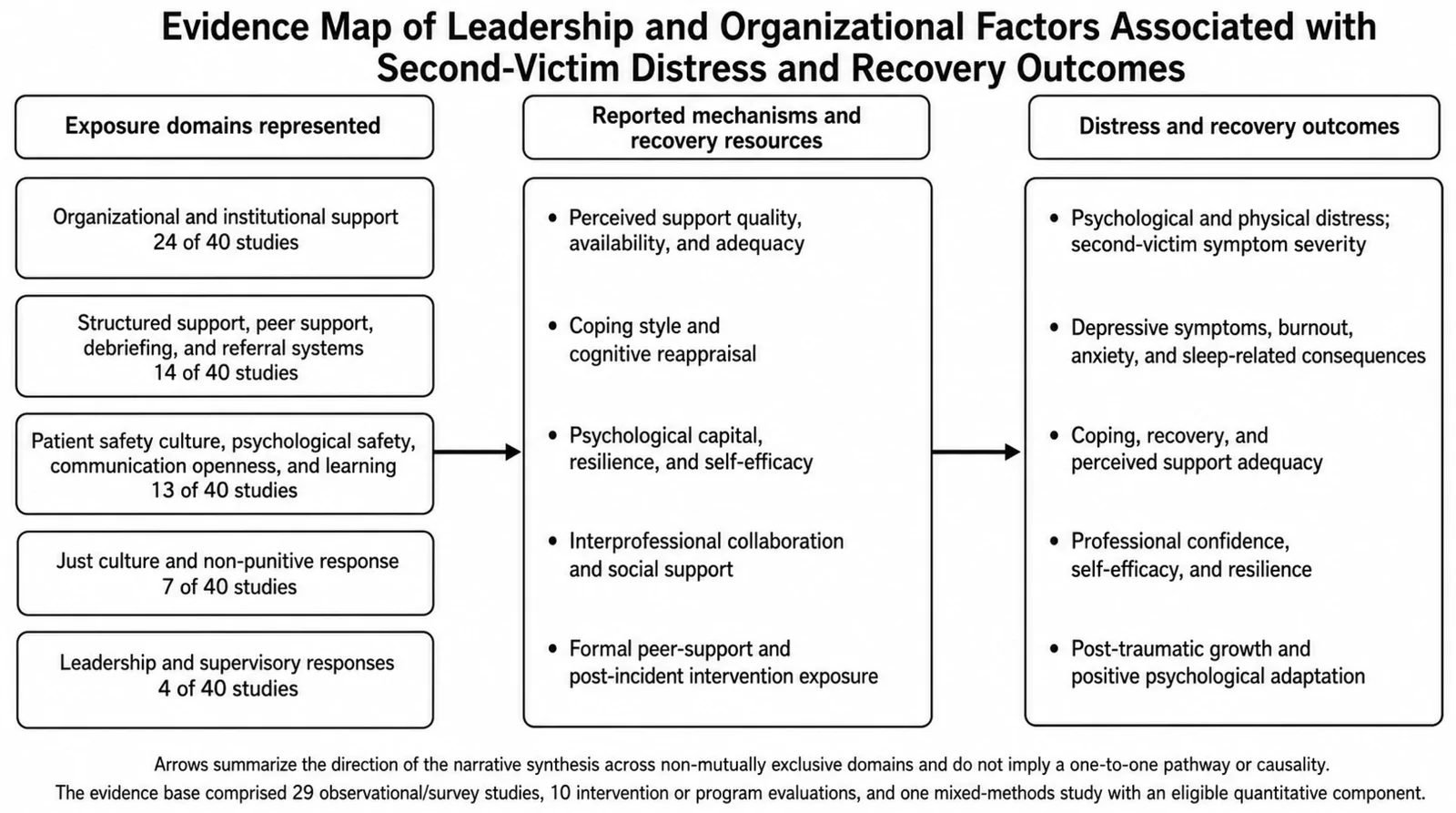
Evidence map of leadership-related and organizational exposure domains, reported recovery mechanisms, and second-victim distress or positive-adaptation outcomes across the 40 studies contributing to the distress/recovery domain. Exposure-domain counts are non-mutually exclusive. Arrows summarize the direction of the narrative synthesis and should not be interpreted as one-to-one pathways or causal effects.

Figure 3 summarizes the exposure domains, reported recovery mechanisms, and distress or positive-adaptation outcomes represented in this evidence base.

### 3.5. Professional Consequences and Work-Related Outcomes

Professional consequences were examined in 21 of the 44 included studies (47.7%). Within this subset, organizational and institutional support was represented in 13 studies, patient safety culture, psychological safety, communication openness, or organizational learning in seven, just culture or non-punitive response in six, structured second-victim support or related post-incident pathways in three, and leadership or supervisory responses in two; these exposure domains were non-mutually exclusive. Nineteen studies used observational or survey-based designs, one evaluated a system-level peer-support program, and one used a mixed-methods design with an eligible quantitative component.

Turnover intention and absenteeism were the most consistently investigated workforce outcomes. Across studies, greater second-victim distress was generally associated with more negative work-related outcomes, whereas perceived organizational, colleague, or supervisor support was associated with lower turnover-related responses, absenteeism, or job insecurity [25,27,30,31,75,79]. Several mediation and path models suggested that organizational support or safety culture may relate to work outcomes partly through second-victim distress or support-related pathways, but the cross-sectional nature of these models did not establish temporal sequencing [23,24,25,27,30].

Safety-culture and just-culture findings were directionally consistent with fewer negative professional consequences in several settings [22,23,24,45]. Kim et al. [45] distinguished constructive from defensive changes in clinical practice and identified cross-sectional modeled pathways linking just culture, second-victim experience, coping, and defensive practice. Defensive practice was therefore retained as a professional-consequence outcome rather than interpreted as evidence of improved patient safety.

Other studies extended the evidence to burnout, emotional exhaustion, professional disengagement, organizational attachment, and intention to leave [19,21,33,59,60,70,73,76,77,78,79]. Although the overall direction was consistent with a potentially protective association of supportive organizational conditions, the studies used heterogeneous instruments and often measured intentions or perceptions rather than objectively verified workforce events.

Overall, professional-consequence findings were directionally coherent but remained predominantly cross-sectional and self-reported. No included study prospectively verified actual staff turnover or administrative absence over time, and program-level evidence was limited. The available evidence therefore supports organizationally modifiable associations and plausible pathways, but not definitive claims that any specific leadership response or support program prevents workforce loss. Detailed estimates are retained in Table 4.

### 3.6. Structured Support Programs and Implementation Outcomes

Eleven included studies evaluated a structured post-incident intervention or support program [36,37,39,42,63,64,65,66,67,69,70]. Seven examined internal peer-support services or formal peer-support networks [36,37,39,42,65,66,70], two evaluated mindfulness-based interventions [64,69], one assessed an external psychological support program [67], and one evaluated a restorative just-culture incident-response framework that incorporated staff support, stakeholder engagement, and organizational learning [63]. Program awareness, reach, uptake, utilization, satisfaction, and perceived usefulness were summarized as implementation outcomes. Consistent with the prespecified synthesis plan, these measures were not interpreted as evidence of improved incident reporting, disclosure, safety behavior, safety performance, or patient outcomes unless an eligible outcome had been measured directly.

The most consistent implementation signal was improved awareness of support and perceived availability of organizational resources. In pediatric inpatient and intensive-care services, Finney et al. found that awareness of the term “second victim” increased from 51.8% before implementation to 74.0% afterward (*p* < 0.001), while the proportion of respondents who considered support resources adequate increased from 35.8% to 89.1% (*p* < 0.001). After implementation, 65.7% indicated that they would be likely to use the program themselves and 84.6% would use it to support a colleague [36]. Two years after implementation of a departmental anesthesia peer-support program, psychological distress was lower, and more than 80% of respondents expressed favorable views of the program and its effect on safety culture [39]. In women’s services, Webb et al. trained 26 peer supporters across four departments and both shifts; the proportion of respondents reporting that they had received support after an adverse event increased from 43% before implementation to 86% afterward [65].

Program reach and actual utilization were reported less consistently than awareness. Thompson et al. trained eight volunteer certified registered nurse anesthetists to provide peer support continuously, with access through self-identification or referral by a colleague or leader [37]. The external YANA program attracted 26 applicants, but only 11 (42.3%) completed all three sessions and 10 (38.5%) completed both assessments. Participants generally reported high satisfaction, willingness to recommend the program, and willingness to participate again; however, the 57.7% attrition rate demonstrated the difficulty of engaging affected clinicians even when support was offered outside their employing institution [67]. The largest implementation dataset came from the children’s healthcare system evaluated by Fall et al., in which 331 referrals were recorded: 31% were self-initiated, 28% originated from an organizational leader, and 23% from the quality-and-safety team. Adverse patient events or outcomes accounted for 48% of referrals, and 58% of referrals resulted in peer support being provided [70].

Where participant-level outcomes were assessed, findings were generally favorable but derived from small or non-randomized evaluations. Li et al. reported an increase in mean mindfulness-awareness scores from 64.85 ± 11.41 before the intervention to 68.63 ± 11.33 at week 4 and 71.20 ± 8.41 at week 8 (F = 18.848, *p* < 0.001). Work-related organizational-support needs also changed over time (F = 34.513, *p* < 0.001), with support preferences shifting from withdrawal toward discussion with respected peers and senior colleagues [64]. In the external support study, median IES-R-K scores decreased from 30.0 to 16.0 among the 10 paired participants (*p* = 0.028; r = 0.65) [67]. In a pediatric intensive-care unit, distress decreased significantly after 13 encounters with trained peer supporters (t(12) = 16.40, *p* < 0.001) [42].

Chen et al. provided the only controlled intervention comparison. Among intensive-care nurses, the experimental group’s mean Pittsburgh Sleep Quality Index score decreased from 12.94 ± 2.03 to 9.32 ± 2.13, compared with a change from 12.76 ± 2.21 to 10.72 ± 1.99 in the control group. The intervention was also associated with more favorable resilience and perceived-support scores. The authors reported a reduction in adverse-event cases from 50 before the intervention to 33 afterward; because this was a retrospective case–control evaluation and the event-count analysis was not based on randomized allocation or a prespecified incidence denominator, the finding was treated as a preliminary safety indicator rather than definitive evidence of reduced patient harm [69].

Program evaluations also suggested possible organizational effects. After implementation of a skilled peer-support program in anesthesiology and perioperative services, 62.5% of respondents stated that institutional support had improved since their serious adverse event. Formal emotional-support availability increased significantly (*p* = 0.02), and post-implementation respondents had higher adjusted odds of perceiving that the organization had learned from the event to prevent recurrence (aOR = 3.9, 95% CI 1.01–15.1) [66]. Fall et al. found that staff who experienced distress after harm events and were offered peer support reported lower burnout than those not offered support (63% vs. 75%, *p* < 0.01) and more favorable overall and local-unit safety-culture ratings [70]. These comparisons were observational and remain vulnerable to referral, response, and selection bias.

The restorative just-culture framework evaluated by Turner et al. extended support beyond an individual peer-support encounter by integrating staff support, consumer and family involvement, structured review, open disclosure, and organizational learning. In all 19 post-implementation incident reviews, staff were offered support. Reviews produced recommendations after every incident, compared with 78.1% before implementation (*p* = 0.010), and the mean number of recommendations increased from 1.9 to 3.9 per incident (*p* = 0.001). Clinician disclosure was offered in 17 of 19 incidents and occurred in 15; these were descriptive process indicators and were not classified as an exposure-linked reporting or disclosure outcome [63].

Overall, structured support programs appeared feasible across pediatric, anesthesia, perioperative, intensive-care, mental-health, and multispecialty settings. Awareness, perceived resource availability, satisfaction, and immediate acceptability improved more consistently than objectively verified clinical or workforce outcomes. Implementation was facilitated by trained peers, confidential and readily accessible contact routes, leadership and quality-team referrals, and integration with incident-response systems. Recurrent barriers included low participation or retention, uncertain knowledge of how to access supporters, reluctance to document confidential encounters, small evaluation samples, and inconsistent reporting of program reach and fidelity. Only one study included a comparison group, and that comparison was retrospective and non-contemporaneous. Consequently, these studies support the feasibility and acceptability of structured support but provide limited certainty regarding sustained recovery, workforce retention, reporting behavior, or patient-safety effects. Table 5 summarizes the program models, implementation indicators, eligible outcome signals, and principal interpretive limitations.

### 3.7. Critical Appraisal and Methodological Limitations of the Included Studies

The design-specific JBI appraisal included 34 analytical cross-sectional studies, nine quasi-experimental or uncontrolled program evaluations, and one retrospective case–control evaluation. Across the cross-sectional evidence, eligibility criteria, study settings, outcome measurement, and statistical methods were generally reported adequately. However, the concurrent measurement of organizational exposures, potential mediators, and outcomes prevented confirmation of temporal ordering. Reliance on self-reported measures, convenience or voluntary recruitment, and possible residual confounding further limited causal interpretation.

The quasi-experimental evidence was consistently limited by the absence of concurrent control groups and by the lack of multiple measurements before and after implementation. Several evaluations also had small samples, incomplete follow-up, unmatched pre–post respondents, or insufficient reporting of program exposure, reach, and fidelity. The single case–control evaluation used non-contemporaneous comparison groups, increasing susceptibility to selection bias, secular changes, and residual confounding.

Accordingly, the appraisal did not identify a sufficient basis for strong causal conclusions regarding the effectiveness of any specific leadership response or structured support program. The evidence was considered most informative for identifying consistent associations, plausible organizational pathways, program feasibility, and short-term implementation signals. Complete item-level JBI judgments and study-specific appraisal considerations are reported in Supplementary Table S2.

### 3.8. Overall Synthesis of the Main Findings and Evidence Gaps

Across the 44 included studies, the evidence demonstrated a clear hierarchy of outcome coverage. Leadership-related and organizational factors were examined predominantly in relation to second-victim distress and recovery (40/44, 90.9%) and, to a lesser extent, professional consequences (21/44, 47.7%). One study (2.3%) evaluated disclosure-related behavior, five studies (11.4%) evaluated other safety-related outcomes or indicators, no study prospectively evaluated incident or near-miss reporting or speaking up, and directly measured patient-safety outcomes remained sparse. The literature therefore concentrated on clinician well-being and workforce functioning rather than on directly measured safety behavior or patient outcomes.

The most consistent cross-study pattern was that more supportive organizational responses, stronger just and non-punitive cultures, learning-oriented safety climates, and accessible structured support were associated with lower distress, more favorable coping and recovery, and fewer negative work-related outcomes. Program evaluations additionally suggested that structured peer support and related post-incident pathways can improve awareness, perceived availability, acceptability, and some short-term psychological outcomes. However, these findings were derived mainly from cross-sectional self-report studies and uncontrolled or non-randomized program evaluations. The JBI appraisal therefore supports an interpretation of organizationally modifiable associations and plausible pathways, but not definitive causal effects.

Confidence differed substantially across outcome domains. The distress/recovery domain contained the largest and most directionally consistent body of evidence, but confidence in causal interpretation remained limited because most contributing studies were cross-sectional, self-reported, and vulnerable to residual confounding or uncertain temporal ordering. Professional-consequence findings were also directionally coherent, but most outcomes reflected self-reported intentions or occupational responses rather than prospectively verified turnover or absence. Confidence in the effectiveness of structured interventions was limited because evaluations were predominantly uncontrolled or non-randomized and were frequently constrained by small samples, incomplete follow-up, or insufficient information on program exposure and fidelity. Confidence regarding reporting/disclosure and other directly measured safety outcomes was lowest because the evidence was sparse and heterogeneous; the disclosure domain relied on a single cross-sectional study with an internally inconsistent description of one effect estimate.

**Figure 4:**
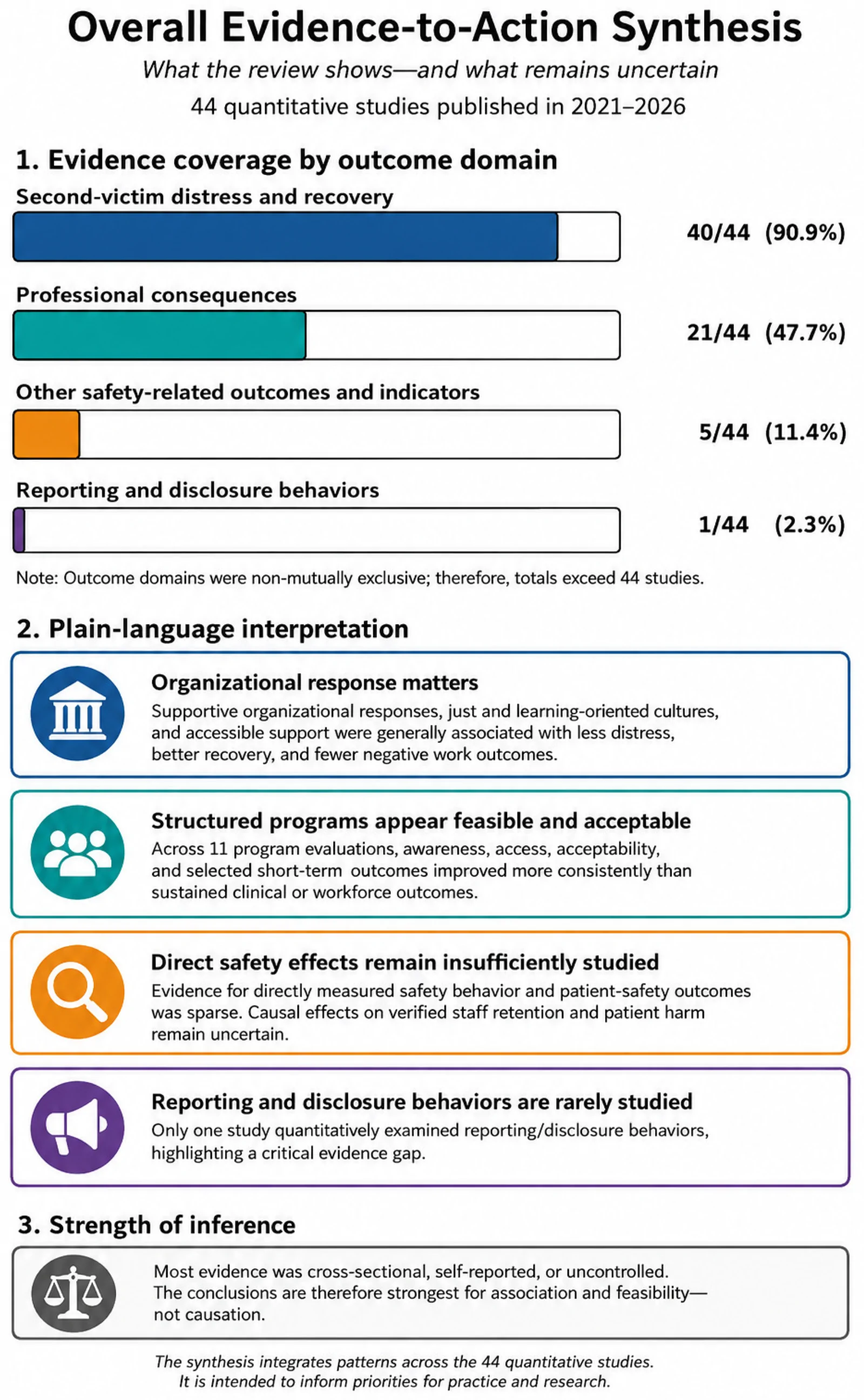
Overall evidence-to-action synthesis of the 44 included studies. Outcome-domain counts are numbers of distinct studies and were not mutually exclusive across the review. The figure distinguishes the volume of evidence from the certainty of causal inference. Associations with distress/recovery and professional outcomes were directionally consistent but were based predominantly on cross-sectional self-report evidence. Evidence for directly measured safety behaviors and patient-safety outcomes was sparse.

Taken together, the review supports a bounded conclusion: organizational response appears relevant to how healthcare professionals experience and recover from patient safety incidents and may also relate to professional functioning. A central evidence gap remains whether these organizational responses change directly measured reporting, disclosure, speaking up, verified workforce outcomes, or patient-safety outcomes. Evidence volume and directional consistency should therefore not be interpreted as evidence of established causal effectiveness. Table 6 summarizes the evidence patterns and principal interpretive constraints across outcome domains. Figure 4 summarizes this evidence-to-action interpretation for clinical leaders, policy-makers, researchers, and non-specialist readers.

## 4. Discussion

### 4.1. Principal Findings

This systematic review synthesized quantitative evidence from 44 studies examining leadership-related and organizational responses following patient safety incidents. The principal finding was that organizational response was directionally associated with how healthcare professionals experience and recover from post-incident distress. More supportive organizational and institutional responses, just and non-punitive cultures, learning-oriented safety climates, and accessible structured support were generally associated with lower distress, more favorable coping and recovery, and fewer negative professional consequences. However, the available evidence primarily supports associations and plausible organizational pathways rather than causal effects.

The strongest and most extensive evidence concerned second-victim distress and recovery. Forty of the 44 included studies assessed outcomes such as psychological or physical distress, coping, resilience, professional self-efficacy, perceived support adequacy, or post-traumatic growth. Across different healthcare systems and professional groups, less favorable organizational support and more punitive or poorly supportive environments were generally associated with more severe symptoms or less favorable recovery-related outcomes. Several mediation, path-analysis, and structural-equation models further suggested that organizational factors may operate through psychological capital, coping style, perceived support, professional efficacy, or second-victim distress. Nevertheless, because most of these variables were measured concurrently, the temporal sequence among organizational exposure, mediator, and outcome could not be established.

Professional consequences constituted the second most frequently examined outcome domain. Twenty-one studies evaluated turnover intention, intention to leave, absenteeism, burnout, job insecurity, defensive practice, or reduced professional functioning. The findings were directionally consistent with the distress-related evidence: stronger organizational support, just culture, patient safety culture, peer support, and recovery resources were associated with fewer negative work-related outcomes. However, most studies measured intentions, perceptions, or self-reported occupational responses rather than objectively verified turnover, sickness absence, or longer-term workforce retention. The evidence therefore indicates potentially modifiable organizational relationships, but it does not establish that a particular leadership response or support intervention prevents workforce loss.

Structured post-incident programs also produced promising but bounded findings. Evaluations of peer-support services, mindfulness-based interventions, external psychological support, and restorative incident-response frameworks generally demonstrated improvements in program awareness, perceived availability of support, acceptability, satisfaction, and some short-term psychological outcomes. These findings support the feasibility of implementing structured support across diverse clinical settings. At the same time, most evaluations were uncontrolled, used small or unmatched samples, had limited follow-up, or incompletely reported program reach, fidelity, and sustained use. Accordingly, the intervention literature should be interpreted as demonstrating feasibility, acceptability, and selected short-term signals rather than established effectiveness.

A major finding was the imbalance between clinician-centered outcomes and directly measured safety outcomes. One included cross-sectional study assessed disclosure-related behavior in relation to patient safety culture, while no study prospectively evaluated incident or near-miss reporting or speaking up as a principal exposure-linked outcome. Only five studies evaluated other safety-related outcomes or patient-safety indicators. Consequently, improvements in perceived support, distress, recovery, program utilization, or safety-culture perceptions cannot be assumed to represent improvements in reporting behavior, clinical safety performance, or patient outcomes. This distinction is central to the interpretation of the review and indicates that organizational support for affected healthcare professionals and demonstrable improvements in patient safety should be treated as related but analytically separate objectives.

The fragility of the safety-behavior evidence is particularly evident in the disclosure domain. Istrate et al. [71] was the only included study to evaluate disclosure-related behavior in relation to an eligible organizational exposure, and the narrative direction reported for one disclosure estimate conflicted with the corresponding odds ratio. We therefore did not interpret that estimate directionally. When a prespecified outcome domain depends on a single cross-sectional study containing an internally inconsistent effect description, neither the direction nor the robustness of the broader association can be established. This further supports the need for prospective studies using clearly defined and independently interpretable disclosure outcomes.

The methodological appraisal reinforced a cautious interpretation. The evidence base was dominated by cross-sectional self-report studies, while most intervention evaluations lacked concurrent controls or repeated pre- and post-intervention measurements. Residual confounding, selection bias, non-randomized designs, and uncertain temporal ordering were recurrent concerns. Taken together, the findings provide a coherent basis for concluding that leadership and organizational responses matter for clinician recovery and professional functioning, but the evidence remains insufficient to determine which specific organizational strategies produce sustained causal improvements in workforce or patient-safety outcomes.

### 4.2. Interpretation in Relation to Existing Evidence

The findings of this review are broadly consistent with previous syntheses of second-victim support while extending them through an outcome-specific quantitative framework. A recent systematic review of interventions for healthcare professionals affected by patient safety incidents identified substantial heterogeneity in intervention content, implementation approach, outcome measurement, and certainty of evidence [47]. The present review reached a similar conclusion: structured peer-support services and other post-incident programs generally improved awareness, perceived accessibility, acceptability, and selected short-term psychological outcomes, but evidence for sustained recovery, workforce retention, or patient-safety effects remained limited. This convergence indicates that the main limitation of the current intervention literature is not the absence of promising organizational models, but the shortage of controlled, longitudinal, and consistently measured evaluations.

The results also support the conclusions of the systematic review by Simms-Ellis et al., which identified a persistent mismatch between the support healthcare professionals want, the support they receive, and the support they perceive as effective following patient safety incidents [48]. In the present synthesis, perceived organizational and institutional support was the most frequently examined exposure domain, and lower support quality or limited access to resources was generally associated with more severe distress and less favorable professional outcomes. Program evaluations further showed that increasing awareness of a service did not necessarily ensure sustained uptake or completion. Attrition, uncertainty about how to contact trained supporters, reluctance to document confidential encounters, and inconsistent referral pathways remained recurrent implementation barriers. These findings suggest that the existence of a formal program is insufficient unless support is visible, trusted, confidential, readily accessible, and integrated into routine incident-response processes.

The present findings are also compatible with qualitative evidence describing reciprocal relationships between individual post-incident experiences and organizational responses [50]. Supportive leadership, fair accountability, communication openness, peer support, and opportunities for organizational learning may shape how an incident is interpreted and whether the affected professional moves toward recovery, withdrawal, defensive practice, or continued occupational distress. Conversely, severe distress and unmet support needs may influence engagement with colleagues, organizational processes, and future clinical work. The quantitative mediation and path models included in this review provide plausible statistical representations of these relationships, particularly through perceived organizational support, coping, psychological capital, professional efficacy, and second-victim distress. However, the predominantly cross-sectional design of these models means that reciprocal or reverse pathways cannot be excluded.

Variation in measurement also helps explain the heterogeneity of the findings. The scoping review of the Second Victim Experience and Support Tool documented differences in validation, adaptation, and application across professional groups and healthcare settings [49]. Similarly, the studies included in the present review used different SVEST versions, distress measures, support constructs, workforce outcomes, and scoring directions. Some studies assessed broad composite dimensions, whereas others evaluated distinct outcomes such as depressive symptoms, professional self-efficacy, turnover intention, burnout, or post-traumatic growth. This measurement diversity limited direct comparison and prevented quantitative pooling. It also reinforces the need to distinguish perceived organizational support, support needs, program implementation measures, psychological recovery, and professional consequences rather than treating them as interchangeable indicators of a single second-victim outcome.

This review adds to the existing literature by specifically examining quantitative relationships between predefined leadership-related or organizational exposures and measurable post-incident outcomes. Previous completed reviews have focused principally on intervention models, support preferences, measurement instruments, or qualitative experiences [47,48,49,50], while a published scoping-review protocol has proposed mapping leadership factors in hospital settings [51]. In contrast, the present synthesis separated leadership and supervisory responses, organizational support, just culture, safety and learning climate, and structured support systems, and examined their relationships across four distinct outcome domains. This approach demonstrated that the most developed evidence concerns clinician distress, recovery, and professional functioning, whereas quantitative evidence concerning direct safety behavior and patient outcomes remains markedly underdeveloped.

The findings are consistent with the broader organizational priorities of the World Health Organization, which emphasize leadership commitment, workforce protection, learning-oriented safety culture, reporting systems, and organizational learning [1]. Nevertheless, the current evidence does not demonstrate that improvements in clinician support automatically translate into safer care. Organizational responses may simultaneously pursue staff recovery, workforce sustainability, learning, and patient safety, but each objective requires its own directly measured outcomes. Evaluations of second-victim initiatives should therefore assess both clinician-centered outcomes and independent safety-related indicators rather than using perceived support, program satisfaction, or safety-culture perceptions as substitutes for observed changes in reporting, clinical practice, or patient harm.

### 4.3. Implications for Leadership, Organizational Policy, and Practice

The available evidence supports consideration of post-incident support as an important organizational responsibility and evidence-informed priority, while the effectiveness of specific leadership or support models remains uncertain. Organizational responses may influence clinician distress, recovery, professional functioning, and willingness to remain in practice, but improvements in clinician-centered outcomes should not be assumed to demonstrate improved safety performance or reduced patient harm.

Frontline supervisors, clinical managers, and senior leaders are often positioned to provide the first organizational response after an incident. Organizations may therefore consider clear procedures for timely acknowledgement, supportive contact, assessment of immediate needs, confidential referral, and appropriate follow-up, together with preparation of managers for non-punitive and psychologically safe responses. However, leadership and supervisory responses were directly examined in only four included studies. Manager-led approaches should consequently be regarded as evidence-informed organizational priorities rather than interventions with established causal effectiveness.

Structured support may be organized within a tiered pathway that includes immediate local support, access to trained peers, and escalation to occupational-health, psychological, or specialist services when required [36,37,39,42,63,64,65,66,67,69,70]. Flexible access routes may include self-referral, referral by colleagues or managers, and activation through quality-and-safety teams. The reviewed evaluations suggest that such models are feasible and often acceptable, but they do not establish that a particular configuration is superior or that implementation produces sustained clinical or workforce benefit.

Implementation is best evaluated beyond whether a program has been introduced. Relevant indicators include awareness, referral sources, reach, uptake, time to contact, completion, attrition, participant satisfaction, and fidelity to the intended support model. The included evaluations showed that high awareness or favorable attitudes did not necessarily result in sustained participation or complete follow-up [36,65,67,70].

The available evidence also supports consideration of fair, just-culture, and learning-oriented approaches to incident management. Non-punitive organizational conditions were associated with lower distress, fewer negative work-related outcomes, and more constructive practice-related responses in several studies [22,23,24,45]. Such findings support the importance of distinguishing fair accountability and system learning from blame-oriented responses, while remaining insufficient to establish the causal effectiveness of a specific just-culture intervention.

Evaluation frameworks should distinguish clinician recovery, workforce outcomes, implementation performance, and patient safety. Distress, coping, perceived support, burnout, turnover intention, and program satisfaction are important outcomes, but they should not be used as substitutes for incident reporting, speaking up, observed safety behavior, verified staff retention, clinical outcomes, or patient harm.

Finally, because the current evidence was concentrated in hospital settings and nursing populations, these organizational priorities should not be assumed to transfer unchanged to primary care, community or prehospital services, or to underrepresented medical, surgical, allied-health, pharmacy, and other professional groups. Local adaptation should be accompanied by prospective evaluation before equivalent effects are inferred in these settings.

### 4.4. Strengths and Limitations

This review has several methodological strengths. The review question, PECOS framework, eligibility criteria, exposure categories, outcome domains, and planned synthesis approach were prespecified and registered in PROSPERO. Study identification combined two primary bibliographic databases with two supplementary open-index sources and forward citation searching. Screening, full-text eligibility assessment, and data extraction were conducted independently by two reviewers. Design-specific JBI critical-appraisal judgments were reviewed and agreed by two reviewers, with disagreements resolved through consensus. Reporting followed PRISMA 2020 and PRISMA-S, the narrative synthesis was informed by SWiM, and methodological appraisal used the relevant JBI instruments without applying an arbitrary composite score or exclusion threshold [53,54,55,56].

A further strength was the outcome-specific organization of the synthesis. Reporting and disclosure behaviors, other safety-related outcomes, second-victim distress and recovery, and professional consequences were examined separately rather than combined into a single broad outcome category. Leadership-related and organizational factors were similarly classified into five non-mutually exclusive exposure domains. This framework reduced the risk of interpreting improvements in perceived support, distress, program awareness, or satisfaction as evidence of improved safety behavior or reduced patient harm. Implementation measures were also summarized separately from eligible clinical, professional, and safety-related outcomes.

Several review-level limitations should nevertheless be considered. The review was restricted to English-language publications within a prespecified contemporary evidence window beginning in 2021. Given the international distribution of second-victim research, the English-language restriction may have introduced language and selection bias and may have reduced representation of some healthcare systems and cultural contexts. The omission of CINAHL, Embase, and APA PsycINFO represents an additional important limitation. Although study identification combined PubMed and Scopus with supplementary searching in Europe PMC and OpenAlex, two complementary high-sensitivity search approaches, and forward citation searching in Scopus and the Web of Science Core Collection, these methods cannot be assumed to reproduce the unique indexing coverage of discipline-specific nursing, psychological, or biomedical databases. Relevant studies may therefore have been missed, and the magnitude of this potential retrieval limitation cannot be quantified. Grey literature, dissertations, conference abstracts, and unpublished evaluations were not eligible, and publication or dissemination bias therefore cannot be excluded. Although forward citation searching did not identify any additional eligible studies, the total number of citation-searching records was not retained separately.

The characteristics of the included evidence also limited the conclusions. Most studies were cross-sectional, relied on self-reported exposures and outcomes, and recruited participants through convenience, voluntary, or non-probability approaches. Organizational conditions, potential mediators, and outcomes were frequently measured at the same time, preventing confirmation of temporal ordering and allowing the possibility of reverse associations. Adjustment for confounding varied substantially, and unmeasured factors such as incident severity, professional role, prior mental health, organizational context, and access to support may have influenced the reported relationships. Several mediation, path-analysis, structural-equation, and latent-profile studies identified plausible organizational pathways, but these models did not establish causality.

The intervention evidence was similarly constrained. Most program evaluations used uncontrolled pre–post designs, repeated cross-sectional samples, small convenience samples, or short follow-up periods. Only one study included a comparison group, and that comparison was retrospective and non-contemporaneous. Program exposure, implementation fidelity, actual utilization, attrition, and longer-term outcomes were inconsistently reported. Favorable changes in awareness, perceived resource availability, satisfaction, or immediate distress therefore cannot be attributed confidently to the intervention itself or assumed to persist over time.

Clinical and methodological heterogeneity prevented meta-analysis. Studies differed in professional groups, healthcare settings, incident definitions, organizational exposures, measurement instruments, assessment periods, analytical models, and effect measures. A structured narrative synthesis was therefore more appropriate, but narrative synthesis remains vulnerable to selective emphasis and provides no pooled estimate of effect. Formal assessment of reporting bias and formal certainty-of-evidence grading were not undertaken because the studies within the exposure–outcome groupings were insufficiently comparable. Confidence in the findings was instead considered narratively using study design, JBI judgments, consistency, precision, and applicability.

Finally, generalizability was limited by the concentration of evidence in hospital settings and nursing populations. Primary care, community services, prehospital care, pharmacy, allied health, and several medical and surgical specialties were underrepresented. Accordingly, the applicability of the findings is greatest to the hospital-based and nursing contexts represented in the included evidence, and equivalent associations or intervention effects should not be assumed in other professional or organizational settings. Local adaptation and prospective evaluation are required before broader transferability can be established. The 2026 evidence reflects only publications available through the final search date and not a complete calendar year.

### 4.5. Research Priorities

Future research should move beyond predominantly cross-sectional associations and uncontrolled program evaluations. Prospective longitudinal studies are needed to establish the temporal sequence between organizational response, second-victim distress, recovery processes, and professional outcomes. Where feasible, controlled pragmatic trials or well-designed quasi-experimental studies should evaluate structured support programs using concurrent comparison groups, repeated measurements before and after implementation, and sufficiently long follow-up to determine whether early improvements are sustained.

Greater consistency in outcome measurement is also required. Studies should clearly distinguish organizational exposures, psychological recovery outcomes, professional consequences, implementation outcomes, and patient-safety outcomes. Development and adoption of a core outcome set would improve comparability across studies and reduce reliance on heterogeneous composite measures. Such a set should include validated measures of distress and recovery, professional self-efficacy, burnout, turnover intention, verified workforce retention or absence, program reach and fidelity, and independently measured safety-related outcomes.

A particularly important priority is the direct evaluation of safety behavior and patient-safety effects. Future studies should measure incident and near-miss reporting, speaking up, disclosure processes, participation in organizational learning, constructive changes in practice, repeated adverse events, and validated patient-safety indicators. These outcomes should be assessed independently rather than inferred from perceived support, program satisfaction, safety-culture ratings, or reductions in clinician distress. Where patient harm or adverse-event rates are examined, studies should use clearly defined denominators, prespecified observation periods, and appropriate comparison groups.

Research should also examine the mechanisms through which leadership and organizational responses may influence outcomes. Longitudinal mediation models could test whether perceived support, psychological safety, coping, psychological capital, professional efficacy, or access to structured support precede and explain changes in distress, recovery, and professional functioning. Such analyses should avoid treating statistically modeled indirect effects from cross-sectional data as evidence of temporal or causal mechanisms.

Implementation research should assess not only whether a program was introduced, but how it was delivered and used. Future evaluations should report eligibility for support, referral sources, reach, uptake, time to first contact, completion, attrition, fidelity, confidentiality arrangements, escalation pathways, and resource requirements. Mixed-methods process evaluations may help explain why some affected professionals engage with support while others decline, withdraw, or remain unaware of available services. Economic evaluations are also needed to determine the organizational costs of implementation and the potential consequences for absenteeism, turnover, and workforce sustainability.

Finally, the evidence base should be broadened beyond hospital nursing populations. Future studies should include physicians, pharmacists, allied-health professionals, trainees, emergency medical personnel, primary-care teams, community services, and non-hospital settings. Comparative studies across professions, clinical environments, incident types, career stages, and healthcare systems would help determine whether organizational responses require local adaptation and whether particular support models are more effective for specific groups. Multicenter and multinational studies using harmonized definitions and outcome measures would strengthen generalizability and support more reliable translation into policy and practice.

## 5. Conclusions

This systematic review indicates that leadership and organizational responses following patient safety incidents are directionally associated with clinician recovery and professional functioning. Across 44 quantitative studies, supportive organizational and institutional responses, just and non-punitive cultures, learning-oriented safety climates, and accessible structured support were generally associated with lower second-victim distress, more favorable coping and recovery, and fewer negative work-related consequences. Structured support programs appeared feasible and generally acceptable and produced selected favorable short-term signals, but the available evaluations did not establish the effectiveness of specific support models.

A central finding is the marked imbalance between clinician-centered and safety-centered evidence. The field has measured how healthcare professionals experience and recover from patient safety incidents far more extensively than whether organizational responses subsequently alter reporting, disclosure, speaking up, clinical safety behavior, or patient outcomes. Only one included study evaluated disclosure-related behavior, and no study prospectively evaluated incident or near-miss reporting or speaking up as a principal exposure-linked outcome. Improved support, recovery, program uptake, or safety-culture perceptions should therefore not be assumed to translate into safer care unless safety-related outcomes are measured directly.

These conclusions remain bounded by the methodological limitations of the evidence. Most findings were derived from cross-sectional self-report studies or uncontrolled program evaluations, and the available research does not establish the causal effectiveness of any specific leadership response or support model. The applicability of the findings is greatest to hospital-based settings and nursing populations represented in the current evidence base; transferability to primary care, community and prehospital services, and underrepresented professional groups remains uncertain.

The available evidence supports consideration of timely, confidential, tiered, and non-punitive post-incident support as an evidence-informed organizational priority integrated with fair accountability, organizational learning, clear referral pathways, and preparation of managers and peer supporters. Future prospective, longitudinal, and controlled studies should determine which organizational components are effective, for whom, and under which conditions, while separately measuring clinician recovery, verified workforce outcomes, directly measured safety behaviors, and patient-safety outcomes.

## Figures and Tables

**Table 1:** Eligibility criteria based on the PECOS framework.

PECOS Component	Inclusion Criteria	Exclusion Criteria
Population	Practicing healthcare professionals directly or indirectly involved in an unanticipated adverse patient event, healthcare error, patient injury, near miss, or other patient safety incident. Eligible groups included nurses, physicians, pharmacists, midwives, emergency medical personnel, allied healthcare professionals, postgraduate trainees, residents, and multidisciplinary healthcare teams.	Undergraduate or pre-licensure students; individuals exposed only to occupational stress, burnout, workplace violence, or trauma unrelated to a patient safety incident; studies in which data for eligible healthcare professionals could not be distinguished from data for ineligible participants.
Exposure	Leadership-related or organizational factors following patient safety incidents, including supervisor, manager, or senior-leadership responses; perceived organizational or institutional support; just culture; non-punitive responses to error; patient safety culture; psychological safety; communication openness; organizational learning; formal second-victim support systems; structured peer-support programs; post-incident debriefing; and psychological, occupational-health, or professional referral pathways.	Studies that did not assess an eligible leadership-related or organizational exposure; studies examining exclusively individual demographic, personality, coping, or clinical factors without an organizational component.
Comparator	Higher versus lower leadership or organizational support; supportive or just culture versus punitive culture; presence versus absence of a structured support system; intervention versus usual organizational practice; pre-intervention versus post-intervention measurements; or different levels of exposure to organizational support. A formal comparator was not mandatory for eligible observational studies.	Studies without a formal comparator and without a quantitative assessment of the relationship between an eligible exposure and an eligible outcome.
Outcomes	Primary domain: reporting of incidents or near misses, willingness or intention to report, speaking up, and open disclosure. Additional safety-related domain: safety participation, safety performance, engagement in organizational learning, repeated adverse events, and validated patient-safety indicators. Second-victim domain: psychological, physical, or professional distress; symptom severity or duration; coping; recovery; post-traumatic growth; professional confidence; self-efficacy; and perceived adequacy of support. Professional domain: turnover intention, intention to leave clinical practice or the organization, absenteeism, burnout, job satisfaction, organizational commitment, defensive clinical practice, and reduced professional functioning. Eligibility required at least one of the listed outcome domains; assessment of every domain was not required.	Studies reporting none of the prespecified outcomes; studies presenting only general awareness or attitudes without a measurable post-incident outcome; studies in which outcomes could not be linked to a patient-safety incident or an eligible organizational exposure.
Study design	Analytical cross-sectional studies; prospective or retrospective cohort studies; longitudinal studies; case–control studies; quasi-experimental studies; controlled or uncontrolled intervention evaluations; randomized controlled studies; and quantitative components of mixed-methods studies.	Reviews of any type; qualitative-only studies; protocols; editorials; letters; commentaries; conference abstracts; dissertations and theses; validation-only studies without an eligible exposure–outcome analysis; and descriptive prevalence studies without an eligible organizational or leadership factor.

Abbreviations: PECOS, Population, Exposure, Comparator, Outcomes, and Study design.

**Table 2:** PECOS-aligned evidence map of leadership-related and organizational exposure domains and outcome domains across the 44 included studies.

Leadership-Related or Organizational Exposure Domain	Operational Definition	Reporting/Disclosure	Other Safety-Related Outcomes/Indicators	Second-Victim Distress/Recovery	Professional Consequences	Studies in Exposure Domain
Leadership and supervisory responses	Responses, support, and relational resources provided by supervisors, managers, senior leaders, or clinical leaders.	0	0	4	2	4 (9.1%)
Organizational and institutional support	Perceived organizational or institutional support, support quality, resource availability, and supportive organizational response.	0	1	24	13	26 (59.1%)
Just culture and non-punitive response	Restorative or just culture, fair accountability, and non-punitive handling of error.	0	2	7	6	8 (18.2%)
Patient safety culture, psychological safety, communication openness, and organizational learning	Patient-safety culture, psychological safety, speaking-up climate, incident discussion, communication openness, and learning climate.	1	1	13	7	14 (31.8%)
Structured second-victim support, peer support, debriefing, and referral systems	Formal peer-support services, debriefing, counseling, mindfulness-based support, and referral or incident-response pathways.	0	4	14	3	15 (34.1%)

Notes: Cell values are numbers of distinct studies. Exposure and outcome domains were non-mutually exclusive; a study could contribute to more than one row, column, or cell. Row totals count unique studies within each exposure domain and therefore are not equal to the sum of the outcome cells. Outcome definitions: reporting/disclosure = incident or near-miss reporting, speaking up, and open disclosure when evaluated as outcomes; other safety-related outcomes/indicators = safety participation or performance, organizational learning, repeated events, incident-review quality, or practice-related safety behaviors; second-victim distress/recovery = psychological, physical, or professional distress, coping, recovery, resilience, post-traumatic growth, self-efficacy, or adequacy of support; professional consequences = turnover or intention to leave, absenteeism, burnout, job insecurity, defensive practice, or reduced professional functioning. Abbreviations: PECOS, Population, Exposure, Comparator, Outcomes, and Study design.

**Table 3:** Key quantitative findings and interpretive constraints for reporting/disclosure behaviors, other safety-related outcomes, and patient-safety indicators.

Study and Design	Organizational Exposure or Intervention	Safety-Related Outcome	Key Quantitative Finding	Interpretive Constraint
Istrate et al. [71]; cross-sectional survey; Romanian nurses (*n* = 995)	Patient safety culture	Disclosure-related communication after adverse events	12.5% reported informing a patient; stronger safety culture was associated with a greater likelihood of apologizing to patients or families (OR = 1.23, 95% CI 1.14–1.33, *p* < 0.001).	Cross-sectional self-report; disclosure was a secondary outcome, and the direction stated for one informing-patients estimate conflicted with its reported OR.
Kim et al. [45] Cross-sectional PLS-SEM; South Korean clinical nurses (*n* = 461)	Just culture and non-punitive response	Constructive changes in nursing practice after a patient safety incident	Positive indirect pathway from just culture to constructive change through second-victim experience and avoidant coping (B = 0.07, *p* < 0.001); lower just-culture conditions were linked with fewer constructive changes (B = −0.12, *p* < 0.001).	Modeled cross-sectional pathways; temporal and causal ordering cannot be established.
Turner et al. [63] Uncontrolled before-after quality-improvement evaluation; mental-health and specialist service	Restorative just-culture incident-response framework with structured staff support	Incident-review quality, stakeholder inclusion, recommendation number and strength	Recommendations per incident increased from 1.9 to 3.9 (t(56) = −3.47, *p* = 0.001); recommendation strength improved by review-team ratings (chi-square(2) = 7.976, *p* = 0.019) and auditor ratings (chi-square(2) = 6.644, *p* = 0.036); 96.0% vs. 64.8% included an implementation-verification plan (*p* < 0.001).	No concurrent control; pre-implementation process audits were unavailable; 61.3% of post-implementation recommendations remained auditor-rated as weak.
Bursch et al. [66] Pre-post survey evaluation; anesthesiology and perioperative services	Skilled peer-support program	Perceived organizational learning and recurrence prevention after an adverse event	Post-implementation agreement that the organization learned and acted to reduce recurrence: aOR 3.9 (95% CI 1.01–15.1).	Small event-exposed analytical subset, repeated cross-sectional surveys, and a wide confidence interval.
Chen et al. [69] Non-contemporaneous case–control evaluation; ICU nurses (*n* = 50 intervention, *n* = 54 control)	Multicomponent mindfulness-based second-victim support program, including reporting-process and non-punitive-management changes	Reported adverse-event cases	Adverse-event cases in the intervention group decreased from 50 before implementation to 33 after implementation (34% reduction).	Non-concurrent groups, short follow-up, multicomponent intervention, and no comparative effect estimate reported for the event-count reduction.
Fall et al. [70] Program implementation and cross-sectional outcome evaluation; children’s healthcare system	Formal system-wide peer-support network	Overall and local-unit patient-safety-culture ratings	Among distressed staff, those offered peer support reported higher overall safety-culture ratings (3.60 vs. 3.19, *p* < 0.0001) and local-unit ratings (3.68 vs. 3.34, *p* = 0.001).	Non-randomized, self-reported exposure and outcomes; potential selection and residual confounding.

**Table 4:** Key adjusted, latent, mediated, and intervention findings for professional consequences and work-related outcomes.

Study	Leadership/Organizational Exposure	Professional Outcome	Key Quantitative or Modeled Finding	Interpretive Limitation
Burlison et al. [30]	Perceived organizational support	Turnover intention; absenteeism	Organizational support fully mediated distress–turnover and distress–absenteeism relationships (Sobel = 2.11 and 2.40; both *p* < 0.05).	Cross-sectional self-report; outcomes reflected intentions/responses rather than verified turnover or absence.
Mohd Kamaruzaman et al. [25]	Colleague, supervisor, and institutional support	Composite negative work outcomes; resilience	Colleague and supervisor support partially mediated distress–negative-work-outcome pathways; colleague support also mediated professional-efficacy pathways.	Multiple-mediation model from cross-sectional data; temporal order not established.
Shao et al. [31]	Perceived support within latent second-victim profiles	Job insecurity; turnover intention	Profiles with greater support and lower distress had lower adjusted job insecurity and turnover intention.	Latent profiles are sample-dependent and do not demonstrate causal effects of support.
Sun et al. [22]	Patient safety culture and non-punitive response	Turnover intention	Multiple safety-culture dimensions were inversely associated with turnover intention; non-punitive response showed B = −0.278 (*p* < 0.01).	Cross-sectional self-report data; turnover intention rather than verified turnover was measured.
Jeong et al. [24]	Just culture	Composite negative work outcomes	Just culture → distress: β = −0.29; just culture → demand for support: β = −0.65; distress → negative outcomes: β = 0.66; demand for support → negative outcomes: β = 0.18.	Path model fit was acceptable, but all variables were measured concurrently.
Kim et al. [45]	Just culture	Constructive and defensive practice changes	Absence of just culture was associated with fewer constructive changes (B = −0.12) and more defensive changes (B = 0.19); indirect defensive-practice effects through distress (B = −0.24) and distress plus avoidant coping (B = −0.10); all *p* < 0.001.	PLS-SEM estimates are associational and derived from self-reported practice change.
Lee and Lee [23]	Patient safety culture; second-victim support	Negative work outcomes, including turnover and absenteeism	Significant indirect effects of patient safety culture on negative work outcomes were identified through second-victim distress.	Serial mediation was modeled in cross-sectional data; reverse pathways remain possible.
Mahat et al. [27]	Perceived organizational support	Intent to leave; absenteeism; resilience	Organizational support partially mediated the associations of distress with intent to leave and absenteeism; no significant direct relationship with resilience was identified.	Small cross-sectional sample and self-reported outcomes limit generalizability.
Aikawa et al. [75]	Organizational support; just culture; patient safety culture	Turnover intention; absenteeism; resilience	More favorable organizational support and safety culture were associated with lower distress, turnover intention, and absenteeism and with greater resilience (all *p* < 0.05).	National cross-sectional survey found that only 15.7% reported available organizational support.
Fall et al. [70]	Formal peer-support network	Burnout	Among distressed staff, those offered peer support reported lower burnout than those not offered support (63% vs. 75%; *p* < 0.01).	Non-randomized observational comparison; referral and support receipt may reflect selection factors.
Ren et al. [79]	Organizational support; just culture; career resilience	Emotional exhaustion; intention to leave	High intention to leave was reported by 40.0%; second-victim impact and emotional exhaustion increased turnover risk, whereas organizational support and career resilience were protective.	Mixed-methods study with a cross-sectional quantitative component; actual turnover was not observed.

Note: Table 4 includes studies that reported adjusted, latent-profile, mediation, path, structural-equation, or intervention estimates relevant to professional consequences. Remaining studies contributing to this outcome domain are described narratively and listed in Supplementary Table S1. Effect directions follow the original study scales. PLS-SEM, partial least squares structural equation modeling.

**Table 5:** Structured post-incident support programs, implementation outcomes, eligible outcome signals, and interpretive limitations.

Study/Program Model	Setting and Implementation Reach	Implementation Outcomes	Eligible Outcome Signal	Principal Limitation
Finney et al. [36] Internal pediatric peer-support program	Multidisciplinary pediatric inpatient/ICU HCPs; *n* = 194 pre and 177 post.	Awareness: 51.8% to 74.0%; adequate resources: 35.8% to 89.1% (both *p* < 0.001). Intended future use: 65.7% for self and 84.6% for a colleague.	Improved perceptions of available support; symptom outcomes remained heterogeneous.	Independent pre/post survey samples; intended use was not actual utilization.
Thompson et al. [37] CRNA peer-support program	CRNAs; *n* = 51 pre-implementation and *n* = 32 post-implementation; eight volunteer peer supporters were trained to provide 24-h support.	Program successfully established with continuous access and a defined referral route.	Pre–post SVEST scores did not differ significantly; eight peer-support encounters were recorded during the first month.	Independent pre/post samples, short one-month follow-up, no control group, and no statistically significant change in SVEST scores.
Turner et al. [63] Restorative just-culture incident-response framework	Mental-health and specialist service; 19 post-implementation incident reviews audited.	Staff support offered in all 19 reviews; clinician disclosure offered in 17 and completed in 15; broader stakeholder participation.	Recommendations present after 100% vs. 78.1% of incidents (*p* = 0.010); 3.9 vs. 1.9 recommendations per incident (*p* = 0.001).	Service-level before/after evaluation; disclosure indicators lacked an exposure-linked comparator.
Li et al. [64] Mindfulness-based support intervention	Nurses as second victims; *n* = 46; repeated assessments over 8 weeks.	Support preferences shifted from withdrawal toward discussion with peers and senior colleagues.	Mindfulness-awareness: 64.85 to 68.63 to 71.20 (F = 18.848, *p* < 0.001); work-related support needs also changed (F = 34.513, *p* < 0.001).	Single-group intervention without a concurrent comparator.
Pelikan et al. [39] Departmental anesthesia peer-support program	Anesthesia professionals; *n* = 348 pre and 231 two years post implementation.	More than 80% expressed favorable views of the program and its perceived effect on safety culture.	Lower reported psychological distress after implementation.	Unmatched pre/post respondents; program exposure and actual use were not uniform.
Webb et al. [65] Women’s-services peer-support program	Twenty-six peer supporters trained across four departments and both shifts; *n* = 44 pre and 17 post.	Reported receipt of support increased from 43% to 86%; participants considered the program worthwhile.	More staff reported receiving peer support following adverse events.	Small post-implementation sample during COVID-19; peer supporters were reluctant to document encounters.
Bursch et al. [66] Skilled peer-support program	Anesthesiology/perioperative personnel; *n* = 94 pre and 84 post.	Formal emotional-support availability increased (*p* = 0.02); 62.5% reported improved institutional support.	Higher adjusted odds of perceived organizational learning after implementation (aOR = 3.9, 95% CI 1.01–15.1).	Uncontrolled pre/post surveys; wide confidence interval and self-reported organizational outcomes.
Choi et al. [67] External YANA psychological support program	Twenty-six applicants; 11 completed all sessions and 10 completed paired assessments.	High satisfaction and willingness to recommend or reuse the program; completion 42.3%, paired follow-up 38.5%.	Median IES-R-K decreased from 30.0 to 16.0 (*p* = 0.028; r = 0.65).	Very small paired sample and 57.7% attrition; no control group.
Chen et al. [69] Mindfulness-based ICU support program	ICU nurses; 50 intervention and 54 control participants in a retrospective case–control evaluation.	Improved perceived colleague/institutional support and resilience.	PSQI: 12.94 to 9.32 vs. 12.76 to 10.72; adverse-event cases reportedly decreased from 50 to 33.	Non-randomized retrospective comparison; safety count lacked a prespecified incidence denominator.
Davis et al. [42] PICU peer-support team	Thirteen HCP encounters evaluated before and after peer-support contact.	Structured three-tier support model with trained peer-support team access.	Marked reduction in encounter-level distress (t(12) = 16.40, *p* < 0.001).	Very small convenience sample; no control condition or longer-term follow-up.
Fall et al. [70] System-wide formal peer-support network	Three-year experience; 331 referrals. Sources: self 31%, leader 28%, quality/safety 23%; 58% resulted in peer support.	Adverse patient event/outcome accounted for 48% of referrals; program linked with quality-and-safety referral pathways.	Among distressed staff, support offer was associated with lower burnout (63% vs. 75%) and higher safety-culture ratings.	Observational program evaluation; referral and response selection may explain part of the association.

Note: Implementation outcomes were summarized only for studies that also met eligibility through at least one prespecified safety-related, second-victim, or professional outcome. Awareness, reach, uptake, utilization, satisfaction, and perceived usefulness were not interpreted as evidence of improved reporting, disclosure, safety behavior, safety performance, or patient outcomes. CRNA, certified registered nurse anesthetist; HCP, healthcare professional; ICU, intensive-care unit; IES-R-K, Korean Impact of Event Scale-Revised; PICU, pediatric intensive-care unit; PSQI, Pittsburgh Sleep Quality Index.

**Table 6:** SWiM-informed structured summary of evidence patterns and interpretive constraints across outcome domains.

Outcome Domain	No. of Studies	Predominant Designs	Overall Narrative Pattern	Main Constraints	Implication for Interpretation
Reporting/disclosure	1	Cross-sectional	Extremely sparse; one favorable apology association, with an internally inconsistent description of one disclosure estimate.	Single study; self-report; internal reporting inconsistency.	Evidence is insufficient for a reliable domain-level conclusion.
Other safety-related outcomes/indicators	5	Cross-sectional plus heterogeneous intervention/QI designs	Generally favorable but heterogeneous preliminary signals.	Different outcomes and designs; uncontrolled or cross-sectional evidence; limited direct patient outcomes.	Findings are preliminary and hypothesis-generating.
Second-victim distress/recovery	40	Predominantly cross-sectional; some uncontrolled interventions	Generally consistent association of supportive organizational conditions with less distress or more favorable recovery.	Self-report, residual confounding, heterogeneous measures, uncertain temporal ordering, weak controlled evidence.	Largest and most directionally consistent evidence base, but causal interpretation remains limited.
Professional consequences	21	Predominantly cross-sectional	Generally consistent association of support/just culture with fewer negative work-related outcomes.	Intentions and self-reported occupational outcomes rather than verified turnover/absence; cross-sectional pathways.	Directionally coherent, but longitudinal and causal inference remains limited.

## Data Availability

No new data were created or analyzed in this study. Data sharing is not applicable to this article.

## Supplementary Materials

The following supporting information can be downloaded at https://www.mdpi.com/article/10.3390/healthcare14183002/s1, Supplementary File S1, Complete Source-Specific Search Strategies; Supplementary Table S1, Detailed Characteristics, Numbered References, and PECOS Domain Classification of the 44 Included Studies; Supplementary Table S2, Study-Level and Item-Level JBI Critical Appraisal of the 44 Included Studies; Supplementary Table S3, Reports Excluded After Full-Text Assessment, with Primary Reasons for Exclusion; and Supplementary File S2, Completed PRISMA 2020 Checklist.
