# Supplementary material for: Leadership and Organizational Responses After Patient Safety Incidents: A Systematic Review of Second-Victim Outcomes and Evidence Gaps in Safety Behaviors
Source: Healthcare (Basel). 2026 Sep 14;14(18):3002. doi: 10.3390/healthcare14183002 (PMC13606040; doi:10.3390/healthcare14183002)
Supplement: Supplementary file 1 [file healthcare-14-03002-s001.zip › Supplementary File S1.pdf]

Supplementary File S1

Complete Source-Specific Search Strategies

Leadership and Organizational Responses After Patient Safety Incidents: A Systematic Review of Second-Victim Outcomes and Evidence Gaps in Safety Behaviors

Final searches and exports were completed on 30 July 2026. Search strings are reproduced in source-specific syntax.

1. Search approach

Two complementary approaches were used. Search A applied a high-sensitivity second-victim concept without requiring an organizational or outcome term. Search B combined patient-safety-incident terminology with healthcare-professional and leadership-related or organizational concepts to identify potentially eligible studies that did not explicitly use the term “second victim”. No outcome or study-design filter was applied.

PubMed and Scopus were the primary bibliographic databases. Europe PMC and OpenAlex were supplementary open-index sources. Forward citation searching was conducted in Scopus and the Web of Science Core Collection. CINAHL, Embase, and APA PsycINFO were not searched because reproducible institutional access and bibliographic export were not available through the institutional access routes available to the review team. Europe PMC and OpenAlex were used to broaden retrieval and were not treated as equivalent substitutes for the omitted discipline-specific databases.

English-language limits were applied in PubMed, Scopus, and Europe PMC. OpenAlex was searched without a language filter; English-language eligibility was applied during screening. The eligible publication window was 1 January 2021 through the final search date in July 2026. Source-specific date limits are reported below exactly as applied. Records outside the prespecified publication period, or without a usable publication year, were removed before title and abstract screening.

2. Search summary

Source	Search approach	Date	Records retrieved
PubMed	Search A: second-victim concept	30 July 2026	425
PubMed	Search B: incident + professionals + organization/leadership	30 July 2026	2,072
Scopus	Search A: second-victim concept	30 July 2026	515
Scopus	Search B: incident + professionals + organization/leadership	30 July 2026	2,009
Europe PMC	Search A: second-victim concept	30 July 2026	592
Europe PMC	Search B: incident + professionals + organization/leadership	30 July 2026	946
OpenAlex	Supplementary second-victim + leadership/organizational search	30 July 2026	279

Total records retrieved from the seven electronic-search exports: 6,838 (PubMed 2,497; Scopus 2,524; Europe PMC 1,538; OpenAlex 279).

3. PubMed

3.1. PubMed Search A - high-sensitivity second-victim concept

Platform/interface: PubMed, U.S. National Library of Medicine. Search and export date: 30 July 2026. The publication-date limit embedded in both PubMed queries extended through 29 July 2026. Records were exported in NBIB format. Records retrieved: 425.

```
(
"second victim"[tiab]
OR "second victims"[tiab]
OR "second-victim"[tiab]
OR "second victim syndrome"[tiab]
```

```
OR "second-victim syndrome"[tiab]
OR "second victim phenomenon"[tiab]
OR "second-victim phenomenon"[tiab]
OR "second victim experience"[tiab]
OR "second victim experiences"[tiab]
OR "second victim distress"[tiab]
OR SVEST[tiab]
OR "Second Victim Experience and Support Tool"[tiab]
)
AND english[la]
AND 2021/01/01:2026/07/29[dp]
```

3.2. PubMed Search B - incident + professionals + organization/leadership

Records retrieved: 2,072.

```
(
"Patient Safety"[Mesh]
OR "Medical Errors"[Mesh]
OR "Near Miss, Healthcare"[Mesh]
OR "patient safety incident"[tiab]
OR "patient safety incidents"[tiab]
OR "adverse event"[tiab]
OR "adverse events"[tiab]
OR "medical error"[tiab]
OR "medical errors"[tiab]
OR "healthcare error"[tiab]
OR "healthcare errors"[tiab]
OR "health care error"[tiab]
OR "health care errors"[tiab]
OR "clinical error"[tiab]
OR "clinical errors"[tiab]
OR "near miss"[tiab]
OR "near misses"[tiab]
OR "adverse patient event"[tiab]
OR "adverse patient events"[tiab]
OR "unanticipated clinical event"[tiab]
OR "unanticipated clinical events"[tiab]
)
AND
(
"Health Personnel"[Mesh]
OR "healthcare professional"[tiab]
OR "healthcare professionals"[tiab]
OR "health care professional"[tiab]
OR "health care professionals"[tiab]
OR clinician*[tiab]
OR nurse*[tiab]
OR physician*[tiab]
OR doctor*[tiab]
OR pharmacist*[tiab]
OR midwi*[tiab]
OR resident*[tiab]
OR "medical trainee"[tiab]
OR "medical trainees"[tiab]
OR "allied health"[tiab]
OR "emergency medical personnel"[tiab]
)
AND
(
"Leadership"[Mesh]
OR "Organizational Culture"[Mesh]
OR "Social Support"[Mesh]
OR leadership[tiab]
OR leader*[tiab]
OR manager*[tiab]
OR supervisor*[tiab]
OR "organizational support"[tiab]
OR "organisational support"[tiab]
OR "institutional support"[tiab]
OR "managerial response"[tiab]
OR "managerial responses"[tiab]
```

```
OR "leadership response"[tiab]
OR "leadership responses"[tiab]
OR "supervisor response"[tiab]
OR "supervisor responses"[tiab]
OR "just culture"[tiab]
OR "patient safety culture"[tiab]
OR "safety culture"[tiab]
OR "psychological safety"[tiab]
OR "communication openness"[tiab]
OR "nonpunitive response"[tiab]
OR "nonpunitive responses"[tiab]
OR "non-punitive response"[tiab]
OR "non-punitive responses"[tiab]
OR "organizational learning"[tiab]
OR "organisational learning"[tiab]
OR "peer support"[tiab]
OR debrief*[tiab]
OR "support program"[tiab]
OR "support programs"[tiab]
OR "support programme"[tiab]
OR "support programmes"[tiab]
OR "support system"[tiab]
OR "support systems"[tiab]
OR "referral pathway"[tiab]
OR "referral pathways"[tiab]
OR "occupational health"[tiab]
)
AND english[la]
AND 2021/01/01:2026/07/29[dp]
```

4. Scopus

4.1. Scopus Search A - high-sensitivity second-victim concept

Platform/interface: Scopus, Elsevier. Search and export date: 30 July 2026. Limits: publication years 2021-2026 and English language. Records were exported in RIS format.
Records retrieved: 515.

```
TITLE-ABS-KEY(
"second victim"
OR "second-victim"
OR "second victim syndrome"
OR "second-victim syndrome"
OR "second victim phenomenon"
OR "second-victim phenomenon"
OR "second victim experience"
OR "second victim experiences"
OR "second victim distress"
OR SVEST
OR "Second Victim Experience and Support Tool"
)
AND PUBYEAR AFT 2020
AND PUBYEAR BEF 2027
AND LANGUAGE(english)
```

4.2. Scopus Search B - incident + professionals + organization/leadership

Records retrieved: 2,009.

```
TITLE-ABS-KEY(
"patient safety incident"
OR "patient safety incidents"
OR "adverse event"
OR "adverse events"
OR "medical error"
OR "medical errors"
OR "healthcare error"
OR "healthcare errors"
OR "health care error"
OR "health care errors"
```

```
OR "clinical error"
OR "clinical errors"
OR "near miss"
OR "near misses"
OR "adverse patient event"
OR "adverse patient events"
OR "unanticipated clinical event"
OR "unanticipated clinical events"
)
AND TITLE-ABS-KEY(
"healthcare professional"
OR "healthcare professionals"
OR "health care professional"
OR "health care professionals"
OR clinician*
OR nurse*
OR physician*
OR doctor*
OR pharmacist*
OR midwi*
OR resident*
OR "medical trainee"
OR "medical trainees"
OR "allied health"
OR "emergency medical personnel"
)
AND TITLE-ABS-KEY(
leadership
OR leader*
OR manager*
OR supervisor*
OR "organizational support"
OR "organisational support"
OR "institutional support"
OR "managerial response"
OR "managerial responses"
OR "leadership response"
OR "leadership responses"
OR "supervisor response"
OR "supervisor responses"
OR "just culture"
OR "patient safety culture"
OR "safety culture"
OR "psychological safety"
OR "communication openness"
OR "nonpunitive response"
OR "nonpunitive responses"
OR "non-punitive response"
OR "non-punitive responses"
OR "organizational learning"
OR "organisational learning"
OR "peer support"
OR debrief*
OR "support program"
OR "support programs"
OR "support programme"
OR "support programmes"
OR "support system"
OR "support systems"
OR "referral pathway"
OR "referral pathways"
OR "occupational health"
)
AND PUBYEAR AFT 2020
AND PUBYEAR BEF 2027
AND LANGUAGE(english)
```

5. Europe PMC

5.1. Europe PMC Search A - high-sensitivity second-victim concept

Platform/interface: Europe PMC advanced search, European Bioinformatics Institute. Search and export date: 30 July 2026. Records were exported in RIS format.

Records retrieved: 592. The exact exported query included an English-language restriction. Because the resulting export contained records outside the prespecified publication window, date eligibility was enforced before title and abstract screening.

```
TITLE_ABS:(
"second victim"
OR "second victims"
OR "second-victim"
OR "second-victim syndrome"
OR "second victim syndrome"
OR "second-victim phenomenon"
OR "second victim phenomenon"
OR "second victim experience"
OR "second victim experiences"
OR "second victim distress"
OR SVEST
OR "Second Victim Experience and Support Tool"
)
AND LANG:eng
```

5.2. Europe PMC Search B - incident + professionals + organization/leadership

Records retrieved: 946. Limits embedded in the query: English language and first publication date from 2021 through 2026.

```
TITLE_ABS:(
"patient safety incident"
OR "patient safety incidents"
OR "adverse event"
OR "adverse events"
OR "medical error"
OR "medical errors"
OR "healthcare error"
OR "healthcare errors"
OR "health care error"
OR "health care errors"
OR "clinical error"
OR "clinical errors"
OR "near miss"
OR "near misses"
OR "adverse patient event"
OR "adverse patient events"
OR "unanticipated clinical event"
OR "unanticipated clinical events"
)
AND TITLE_ABS:(
"healthcare professional"
OR "healthcare professionals"
OR "health care professional"
OR "health care professionals"
OR clinician*
OR nurse*
OR physician*
OR doctor*
OR pharmacist*
OR midwi*
OR resident*
OR "medical trainee"
OR "medical trainees"
OR "allied health"
OR "emergency medical personnel"
)
AND TITLE_ABS:(
leadership
OR leader*
OR manager*
```

```
OR supervisor*
OR "organizational support"
OR "organisational support"
OR "institutional support"
OR "managerial response"
OR "managerial responses"
OR "leadership response"
OR "leadership responses"
OR "supervisor response"
OR "supervisor responses"
OR "just culture"
OR "patient safety culture"
OR "safety culture"
OR "psychological safety"
OR "communication openness"
OR "nonpunitive response"
OR "nonpunitive responses"
OR "non-punitive response"
OR "non-punitive responses"
OR "organizational learning"
OR "organisational learning"
OR "peer support"
OR debrief*
OR "support program"
OR "support programs"
OR "support programme"
OR "support programmes"
OR "support system"
OR "support systems"
OR "referral pathway"
OR "referral pathways"
OR "occupational health"
)
AND LANG:eng
AND (FIRST_PDATE:[2021 TO 2026])
```

6. OpenAlex

6.1. OpenAlex supplementary search

Platform/interface: OpenAlex web search. Search and export date: 30 July 2026. Publication-year filter: 2021-2026. No language filter was applied in OpenAlex; English-language eligibility was applied during screening. Records were exported in RIS format.

Records retrieved: 279.

```
(
"second victim"
OR "second-victim"
OR "second victim phenomenon"
OR "second victim syndrome"
)
AND
(
leadership
OR leader
OR leaders
OR management
OR manager
OR managers
OR supervisor
OR supervisors
OR "nurse manager"
OR "nurse managers"
OR "clinical leader"
OR "clinical leaders"
OR "organizational support"
OR "organisational support"
```

```
OR "managerial support"
)
```

7. Forward citation searching

Forward citation searching of the included studies and key reviews was conducted in Scopus and the Web of Science Core Collection. Citation-search records underwent the same eligibility assessment as electronically retrieved records. No additional eligible studies were identified. The total number of citation-searching records was not retained separately. Backward reference-list checking was not undertaken in the completed review.

8. Export verification

Record totals were verified directly from the seven raw bibliographic export files. The source totals reproduced the 6,838 records reported in the PRISMA flow diagram.

Search	Raw export file	Records
PubMed Search A	pubmed-secondvict-set.nbib	425
PubMed Search B	pubmed-PatientSaf-set (1).nbib	2,072
Scopus Search A	scopus_export_Jul 30-2026_a5bb5174-66ff-44cf-832a-e2ce6a5b738a.ris	515
Scopus Search B	scopus_export_Jul 30-2026_04763373-9539-48e3-b0fe-d7d6ba8fd50a.ris	2,009
Europe PMC Search A	europepmc.ris	592
Europe PMC Search B	europepmc (1).ris	946
OpenAlex	works-ris-EadQWGGTXJKbuhjDAqKaW5.ris	279

After deduplication, 193 records outside the prespecified publication period or without a usable publication year were removed before title and abstract screening, as reported in the main manuscript and PRISMA flow diagram.
