# Supplementary material for: Leadership and Organizational Responses After Patient Safety Incidents: A Systematic Review of Second-Victim Outcomes and Evidence Gaps in Safety Behaviors
Source: Healthcare (Basel). 2026 Sep 14;14(18):3002. doi: 10.3390/healthcare14183002 (PMC13606040; doi:10.3390/healthcare14183002)
Supplement: Supplementary file 1 [file healthcare-14-03002-s001.zip › Supplementary File S2.pdf]

Supplementary File S2

Completed PRISMA 2020 Checklist

Leadership and Organizational Responses After Patient Safety Incidents: A Systematic Review of Second-Victim Outcomes and Evidence Gaps in Safety Behaviors

Section	Topic	Item #	Checklist item	Location where item is reported
TITLE	Title	1	Identify the report as a systematic review.	Title page.
ABSTRACT	Abstract	2	See the PRISMA 2020 for Abstracts checklist.	Structured Abstract.
INTRODUCTION	Rationale	3	Describe the rationale for the review in the context of existing knowledge.	Section 1, Introduction.
INTRODUCTION	Objectives	4	Provide an explicit statement of the objective(s) or question(s) the review addresses.	Section 1, review question and objective.
METHODS	Eligibility criteria	5	Specify the inclusion and exclusion criteria for the review and how studies were grouped for the syntheses.	Section 2.3 and Table 1; synthesis grouping in Sections 2.5-2.6.
METHODS	Information sources	6	Specify all databases, registers, websites, organisations, reference lists and other sources searched or consulted to identify studies. Specify the date when each source was last searched or consulted.	Sections 2.1-2.2; Supplementary File S1, Sections 1-7. Final electronic searches/exports: 30 July 2026.
METHODS	Search strategy	7	Present the full search strategies for all databases, registers and websites, including any filters and limits used.	Section 2.2; Supplementary File S1, Sections 2-6.
METHODS	Selection process	8	Specify the methods used to decide whether a study met the inclusion criteria of the review, including how many reviewers screened each record and each report retrieved, whether they worked independently, and if applicable, details of automation tools used in the process.	Section 2.4.
METHODS	Data collection process	9	Specify the methods used to collect data from reports, including how many reviewers collected data from each report, whether they worked independently, any processes for obtaining or confirming data from study investigators, and if applicable, details of automation tools used in the process.	Section 2.4.
METHODS	Data items	10a	List and define all outcomes for which data were sought. Specify whether all results compatible with each outcome domain in each study were sought and, if not, how results were selected.	Section 2.5 and Table 1.
METHODS	Data items	10b	List and define all other variables for which data were sought and describe assumptions about missing or unclear information.	Section 2.4.
METHODS	Study risk of bias assessment	11	Specify the methods used to assess risk of bias in included studies, including tools used, number of reviewers, and whether they worked independently.	Section 2.4; Supplementary Table S2. Item-level judgments were reviewed and agreed by two reviewers.
METHODS	Effect measures	12	Specify for each outcome the effect measure(s) used in the synthesis or presentation of results.	Section 2.6.
METHODS	Synthesis methods	13a	Describe the processes used to decide which studies were eligible for each synthesis.	Sections 2.5-2.6; outcome- and exposure-domain grouping.
METHODS	Synthesis methods	13b	Describe any methods required to prepare the data for presentation or synthesis, such as handling missing summary statistics or data conversions.	Sections 2.4-2.6; no unsupported imputation or common-metric conversion.
METHODS	Synthesis methods	13c	Describe any methods used to tabulate or visually display results of individual studies and syntheses.	Section 2.6; Figures 1-4; Tables 2-6; Supplementary Tables S1-S2.
METHODS	Synthesis methods	13d	Describe methods used to synthesize results and provide a rationale. If meta-analysis was performed, describe models and heterogeneity methods.	Section 2.6; structured narrative synthesis, SWiM-informed reporting, and rationale for no meta-analysis.
METHODS	Synthesis methods	13e	Describe methods used to explore possible causes of heterogeneity among study results.	Section 2.6; heterogeneity examined narratively; no formal subgroup analysis or meta-regression.
METHODS	Synthesis methods	13f	Describe any sensitivity analyses conducted to assess robustness of synthesized results.	Not applicable; no sensitivity analysis because no quantitative pooling was undertaken.
METHODS	Reporting bias assessment	14	Describe methods used to assess risk of bias due to missing results in a synthesis.	Section 2.6; no formal statistical/graphical assessment, with rationale reported.
METHODS	Certainty assessment	15	Describe methods used to assess certainty (or confidence) in the body of evidence for an outcome.	Section 2.6; narrative confidence based on study design, JBI judgments, consistency, precision, applicability, and key methodological constraints; no formal certainty grading.
RESULTS	Study selection	16a	Describe the results of the search and selection process, from records identified to studies included, ideally using a flow diagram.	Section 3.1 and Figure 1.
RESULTS	Study selection	16b	Cite studies that might appear to meet inclusion criteria but were excluded, and explain why they were excluded.	Section 3.1; Supplementary Table S3.
RESULTS	Study characteristics	17	Cite each included study and present its characteristics.	Section 3.2; Supplementary Table S1.
RESULTS	Risk of bias in studies	18	Present assessments of risk of bias for each included study.	Section 3.7; Supplementary Table S2.
RESULTS	Results of individual studies	19	For all outcomes, present for each study summary statistics and effect estimates with precision where appropriate.	Sections 3.3-3.6; Tables 3-5; Supplementary Table S1.
RESULTS	Results of syntheses	20a	For each synthesis, briefly summarise characteristics and risk of bias among contributing studies.	Sections 3.3-3.8; Section 3.7; Table 6; Supplementary Table S2.

Section	Topic	Item #	Checklist item	Location where item is reported
RESULTS	Results of syntheses	20b	Present results of all statistical syntheses conducted; if meta-analysis was done, present summary estimates and heterogeneity.	Sections 3.3-3.8; Tables 3-6. No meta-analysis was conducted; findings were synthesized narratively.
RESULTS	Results of syntheses	20c	Present results of all investigations of possible causes of heterogeneity among study results.	Sections 3.3-3.8; possible sources interpreted narratively; no formal subgroup analysis/meta-regression.
RESULTS	Results of syntheses	20d	Present results of all sensitivity analyses conducted to assess robustness of synthesized results.	Not applicable; no sensitivity analyses were conducted.
RESULTS	Reporting biases	21	Present assessments of risk of bias due to missing results for each synthesis assessed.	Not formally assessed; rationale reported in Section 2.6 and discussed as a review-level limitation in Section 4.4.
RESULTS	Certainty of evidence	22	Present assessments of certainty (or confidence) in the body of evidence for each outcome assessed.	Sections 3.7-3.8 and Table 6; Discussion. Narrative interpretive confidence only; no formal certainty grading.
DISCUSSION	Discussion	23a	Provide a general interpretation of the results in the context of other evidence.	Sections 4.1-4.2.
DISCUSSION	Discussion	23b	Discuss any limitations of the evidence included in the review.	Sections 3.7, 4.1, and 4.4; Table 6.
DISCUSSION	Discussion	23c	Discuss any limitations of the review processes used.	Section 4.4.
DISCUSSION	Discussion	23d	Discuss implications of the results for practice, policy, and future research.	Sections 4.3 and 4.5; Section 5, Conclusions.
OTHER INFORMATION	Registration and protocol	24a	Provide registration information for the review, including register name and registration number.	Section 2.1: PROSPERO CRD420261453278.
OTHER INFORMATION	Registration and protocol	24b	Indicate where the review protocol can be accessed, or state that a protocol was not prepared.	Section 2.1: no standalone protocol article was published.
OTHER INFORMATION	Registration and protocol	24c	Describe and explain any amendments to information provided at registration or in the protocol.	Section 2.1: information-source amendment, timing and rationale, no backward citation searching in the completed review, and clarification of publication-type eligibility; updated PROSPERO record.
OTHER INFORMATION	Support	25	Describe sources of financial or non-financial support and the role of funders or sponsors.	Funding statement: no external funding.
OTHER INFORMATION	Competing interests	26	Declare any competing interests of review authors.	Conflicts of Interest statement.
OTHER INFORMATION	Availability of data, code and other materials	27	Report which review materials are publicly available and where they can be found.	Data Availability Statement; Supplementary File S1 and Supplementary Tables S1-S3.

Completed for: Leadership and Organizational Responses After Patient Safety Incidents: A Systematic Review of Second-Victim Outcomes and Evidence Gaps in Safety Behaviors

PRISMA reference: Page MJ, McKenzie JE, Bossuyt PM, et al. The PRISMA 2020 statement: an updated guideline for reporting systematic reviews. BMJ 2021;372:n71. doi:10.1136/bmj.n71.
