# Supplementary material for: Leadership and Organizational Responses After Patient Safety Incidents: A Systematic Review of Second-Victim Outcomes and Evidence Gaps in Safety Behaviors
Source: Healthcare (Basel). 2026 Sep 14;14(18):3002. doi: 10.3390/healthcare14183002 (PMC13606040; doi:10.3390/healthcare14183002)
Supplement: Supplementary file 1 [file healthcare-14-03002-s001.zip › Supplementary Table S1.pdf]

Supplementary Table S1

Detailed characteristics, numbered references, and PECOS domain classification of the 44 included studies

Leadership and Organizational Responses After Patient Safety Incidents: A Systematic Review of Second-Victim Outcomes and Evidence Gaps in Safety Behaviors

Study IDs follow chronological order by final bibliographic publication year. Exposure and outcome domains were non-mutually exclusive. Reference numbers correspond to the main manuscript bibliography.

Study ID / manuscript reference	Country/setting	Population/sample	Design	Organizational exposure domain(s)	Outcome domain(s)	DOI/stable ID
S01 [30] - Burlison et al. (2021)	NR; direct patient-care setting	Nurses; n = 155	Analytical cross-sectional study	Organizational and institutional support	Second-victim distress and recovery; Professional consequences	10.1097/PTS.0000000000000301
S02 [36] - Finney et al. (2021)	NR; pediatric inpatient and intensive-care units	Multidisciplinary pediatric HCPs; n = 194 pre, 177 post	Uncontrolled pre-post peer-support program evaluation	Structured second-victim support, peer support, debriefing, and referral systems	Second-victim distress and recovery	10.1016/j.pedn.2021.08.023
S03 [57] - Nijs et al. (2021)	Belgium; anaesthesia departments	Belgian anaesthesiologists and trainees; n = 456 total respondents, of whom n = 336 reported a patient safety incident during the previous year.	Analytical cross-sectional study	Organizational and institutional support; Safety culture, psychological safety, communication openness, and organizational learning	Second-victim distress and recovery	10.1093/intqhc/mzab058
S04 [20] - Choi et al. (2022)	South Korea; hospital nursing settings	Staff nurses directly involved in PSIs; n = 378	Analytical cross-sectional study with multivariable/path/latent modeling	Organizational and institutional support; Structured second-victim support, peer support, debriefing, and referral systems	Second-victim distress and recovery	10.1111/jan.15221
S05 [58] - Eslami et al. (2022)	Iran; ICU and emergency departments	Nurses; n = 307	Analytical cross-sectional study	Organizational and institutional support; Safety culture, psychological safety, communication openness, and organizational learning	Second-victim distress and recovery; Professional consequences	10.61186/jgbfnm.19.2.10
S06 [59] - Gupta et al. (2022)	United States; national online professional network	U.S. multispecialty physician respondents; n = 1,808 total; n = 1,372 reported adverse-event involvement; n = 1,292 reported emotional impact.	Analytical cross-sectional online survey.	Organizational and institutional support	Professional consequences	10.1097/PTS.0000000000001008
S07 [60] - Hinkley et al. (2022)	United States; 12 professional nursing associations	Registered nurses; n = 1167	Analytical cross-sectional study with multivariable/path/latent modeling	Organizational and institutional support; Structured second-victim support, peer support, debriefing, and referral systems	Second-victim distress and recovery; Professional consequences	10.1111/jnu.12715
S08 [61] - Huang et al. (2022)	China; six tertiary comprehensive hospitals	Nurses involved in at least one adverse event; n = 2897	Analytical cross-sectional study	Safety culture, psychological safety, communication openness, and organizational learning	Second-victim distress and recovery	10.1111/jonm.13490
S09 [62] - Kim et al. (2022)	South Korea; 41 hospital departments	Nurses; n = 296	Analytical cross-sectional study with multivariable/path/latent modeling	Organizational and institutional support; Just culture and non-punitive response; Safety culture, psychological safety, communication openness, and organizational learning	Second-victim distress and recovery	10.1097/QMH.0000000000000330
S10 [19] - Mallea Salazar et al. (2022)	Chile; public and private health institutions	Healthcare workers; n = 301 total; 39.2% reported involvement in an adverse event, and 73.0% of those reported second-victim effects.	Analytical cross-sectional study	Organizational and institutional support	Second-victim distress and recovery; Professional consequences	10.1016/j.jhqr.2021.09.002
S11 [25] - Mohd Kamaruzaman et al. (2022)	Malaysia; three tertiary-care hospitals	Healthcare providers; n = 733	Analytical cross-sectional study	Leadership and supervisory responses; Organizational and institutional support	Second-victim distress and recovery; Professional consequences	10.3390/ijerph19116454
S12 [37] - Thompson et al. (2022)	NR; anaesthesia service	CRNAs; n = 51 pre-implementation and n = 32 post-implementation; 8 peer supporters trained.	Uncontrolled pre-post program evaluation	Organizational and institutional support; Structured second-victim support, peer support, debriefing, and referral systems	Second-victim distress and recovery	10.1016/j.jopan.2021.05.005
S13 [63] - Turner et al. (2022)	Gold Coast mental-health and specialist service	Staff and incident-review stakeholders; n NR	Quality-improvement program evaluation	Just culture and non-punitive response; Structured second-victim support, peer support, debriefing, and referral systems	Other safety-related outcomes and indicators; Second-victim distress and recovery	10.5430/jha.v11n2p8
S14 [64] - Li et al. (2023)	China; comprehensive tertiary hospital in Wuhan	Nurses as second victims; n = 46	Uncontrolled mindfulness-based intervention study	Organizational and institutional support; Structured second-victim support, peer support, debriefing, and referral systems	Second-victim distress and recovery	10.1177/23779608231178136
S15 [39] - Pelikan et al. (2023)	NR; departmental anaesthesia service	Anaesthesia professionals; n = 348 pre, 231 post	Uncontrolled pre-post program evaluation	Organizational and institutional support; Structured second-victim support, peer support, debriefing, and referral systems	Second-victim distress and recovery	Scopus EID 85173053606
S16 [31] - Shao et al. (2023)	China; 25 hospitals across 13 provinces	Nurses directly involved in PSIs; n = 1298	Analytical cross-sectional study with multivariable/path/latent modeling	Organizational and institutional support	Second-victim distress and recovery; Professional consequences	10.1002/nur.22313

Study ID / manuscript reference	Country/setting	Population/sample	Design	Organizational exposure domain(s)	Outcome domain(s)	DOI/stable ID
S17 [22] - Sun et al. (2023)	China; hospitals of different levels in Shandong	Clinical nurses; n = 1525	Analytical cross-sectional study	Just culture and non-punitive response; Safety culture, psychological safety, communication openness, and organizational learning	Second-victim distress and recovery; Professional consequences	10.1002/nop2.1936
S18 [65] - Webb et al. (2023)	NR; women's services in a 350-bed hospital	Nurses; n = 44 pre, 17 post	Uncontrolled pre-post program evaluation	Structured second-victim support, peer support, debriefing, and referral systems	Second-victim distress and recovery	10.5430/jnep.v13n6p49
S19 [66] - Bursch et al. (2024)	NR; three hospitals and six outpatient surgery centres	Anaesthesiology/perioperative personnel; n = 94 pre, 84 post	Uncontrolled pre-post peer-support program evaluation	Organizational and institutional support; Structured second-victim support, peer support, debriefing, and referral systems	Other safety-related outcomes and indicators; Second-victim distress and recovery	10.1016/j.jcjq.2024.03.006
S20 [67] - Choi et al. (2024)	South Korea; three advanced general hospitals and community recruitment	Nurses with PSI experience; n = 26 applicants, n = 11 completed all three program sessions, and n = 10 completed paired pre/post assessments.	Uncontrolled pre-post program evaluation	Structured second-victim support, peer support, debriefing, and referral systems	Second-victim distress and recovery	10.1186/s12912-024-02285-1
S21 [21] - Scarpis et al. (2024)	Italy; five local health authorities in Friuli-Venezia Giulia	Healthcare workers; n = 733 (305 with an adverse event)	Analytical cross-sectional study with multivariable/path/latent modeling	Organizational and institutional support	Second-victim distress and recovery; Professional consequences	10.1097/PTS.0000000000001182
S22 [68] - Bexten et al. (2025)	Germany; three national anaesthesiology symposia	Anaesthesiologists; n = 408	Analytical cross-sectional study	Organizational and institutional support	Second-victim distress and recovery	10.1186/s12871-025-03328-z
S23 [69] - Chen et al. (2025)	NR; intensive-care units	ICU nurses; n = 50 intervention, 54 control	Retrospective case-control intervention evaluation	Structured second-victim support, peer support, debriefing, and referral systems	Other safety-related outcomes and indicators; Second-victim distress and recovery	10.1186/s12912-025-03478-y
S24 [42] - Davis et al. (2025)	United States; pediatric intensive-care unit	PICU nurses/HCPs receiving peer support; n = 13	Quality-improvement program evaluation	Structured second-victim support, peer support, debriefing, and referral systems	Second-victim distress and recovery	10.1097/AJN.0000000000000048
S25 [70] - Fall et al. (2025)	NR; children's healthcare system	Healthcare staff; 331 program referrals; survey n NR	Program implementation and cross-sectional outcome evaluation	Safety culture, psychological safety, communication openness, and organizational learning; Structured second-victim support, peer support, debriefing, and referral systems	Other safety-related outcomes and indicators; Professional consequences	10.1177/25160435251341543
S26 [33] - Hakamy et al. (2025)	Saudi Arabia; secondary- and tertiary-care hospitals in 13 regions	Nurses; n = 117	Analytical cross-sectional study	Leadership and supervisory responses	Second-victim distress and recovery; Professional consequences	10.1111/inr.70031
S27 [71] - Istrate et al. (2025)	Romania; national online and onsite recruitment	Nurses; n = 995	Analytical cross-sectional study	Safety culture, psychological safety, communication openness, and organizational learning	Reporting and disclosure behaviors; Second-victim distress and recovery	10.1186/s12912-025-02745-2
S28 [24] - Jeong et al. (2025)	South Korea; tertiary general hospitals	Clinical nurses; n = 183	Analytical cross-sectional study with multivariable/path/latent modeling	Just culture and non-punitive response	Second-victim distress and recovery; Professional consequences	10.1186/s12912-025-02685-x
S29 [45] - Kim et al. (2025)	South Korea; tertiary hospitals nationwide	Clinical nurses with PSI experience; n = 461	Analytical cross-sectional study with multivariable/path/latent modeling	Just culture and non-punitive response	Other safety-related outcomes and indicators; Professional consequences	10.1155/jonm/1587897
S30 [23] - Lee et al. (2025)	South Korea; hospitals	Nurses; n = 208	Analytical cross-sectional study with multivariable/path/latent modeling	Safety culture, psychological safety, communication openness, and organizational learning	Second-victim distress and recovery; Professional consequences	10.1111/jnu.13048
S31 [72] - Liu et al. (2025)	China; tertiary hospitals in Liaoning Province	Nurses; n = 1719	Analytical cross-sectional study with multivariable/path/latent modeling	Leadership and supervisory responses	Second-victim distress and recovery	10.1186/s40359-025-03291-x
S32 [27] - Mahat et al. (2025)	Finland; two university hospitals	Healthcare professionals; n = 149	Analytical cross-sectional study with multivariable/path/latent modeling	Organizational and institutional support; Just culture and non-punitive response	Second-victim distress and recovery; Professional consequences	10.1111/jan.16291
S33 [74] - Rai et al. (2025)	Multinational; international online survey	Neurointerventionalists; n = 413 complete responses	Analytical cross-sectional online survey.	Organizational and institutional support; Structured second-victim support, peer support, debriefing, and referral systems	Second-victim distress and recovery	10.1136/jnis-2025-024288
S34 [15] - Shin et al. (2025)	Republic of Korea; nurses working in general or tertiary hospitals.	Nurses; n = 435	Analytical cross-sectional study	Organizational and institutional support; Safety culture, psychological safety, communication openness, and organizational learning	Second-victim distress and recovery	10.1111/inr.13081
S35 [28] - Zheng et al. (2025)	China; operating-room nurses recruited from three tertiary Grade A hospitals in Shandong Province.	Operating-room nurses; n = 224	Analytical cross-sectional study.	Organizational and institutional support	Second-victim distress and recovery	10.3389/frhs.2025.1527983
S36 [73] - Qiu et al. (2026)	China; six tertiary Grade A hospitals in Shanxi	Nurses affected by patient suicide; n = 425	Analytical cross-sectional study with multivariable/path/latent modeling	Safety culture, psychological safety, communication openness, and organizational learning	Second-victim distress and recovery; Professional consequences	10.1097/PTS.0000000000001415

Study ID / manuscript reference	Country/setting	Population/sample	Design	Organizational exposure domain(s)	Outcome domain(s)	DOI/stable ID
S37 [75] - Aikawa et al. (2026)	Japan; professional societies/organizations	Experienced healthcare professionals; n = 884	Analytical cross-sectional study with multivariable/path/latent modeling	Organizational and institutional support; Just culture and non-punitive response; Safety culture, psychological safety, communication openness, and organizational learning	Second-victim distress and recovery; Professional consequences	10.1186/s12913-026-14251-5
S38 [76] - Heo et al. (2026)	South Korea; online SAFETY-project survey	Clinical nurses with PSI experience in prior 6 months; n = 518	Analytical cross-sectional study	Safety culture, psychological safety, communication openness, and organizational learning	Second-victim distress and recovery; Professional consequences	10.1002/nur.70089
S39 [77] - Kang et al. (2026)	South Korea; general hospital	Clinical nurses with direct/indirect PSI experience; n = 187	Analytical cross-sectional study	Organizational and institutional support	Professional consequences	10.11111/jkana.2025.0058
S40 [78] - Kappes et al. (2026)	Argentina, Chile, Colombia, and Paraguay; ICUs	ICU nurses; n = 762	Analytical cross-sectional study	Organizational and institutional support; Structured second-victim support, peer support, debriefing, and referral systems	Second-victim distress and recovery; Professional consequences	10.4037/ajcc2026681
S41 [17] - Long et al. (2026)	China; four tertiary hospitals in Changsha	Medical staff with prior adverse-event exposure; n = 237	Analytical cross-sectional study	Leadership and supervisory responses; Organizational and institutional support	Second-victim distress and recovery	10.1155/jonm/5142544
S42 [79] - Ren et al. (2026)	China; 10 tertiary general hospitals in Shanxi and Henan	Operating-room nurses; n = 540	Mixed-methods study (eligible quantitative component)	Organizational and institutional support; Just culture and non-punitive response	Second-victim distress and recovery; Professional consequences	10.1186/s12912-026-05115-8
S43 [16] - Siavashi et al. (2026)	Iran; multiple hospitals	Hospital nurses; n = 435	Analytical cross-sectional study	Safety culture, psychological safety, communication openness, and organizational learning	Second-victim distress and recovery	10.1002/nop2.70623
S44 [80] - Zong et al. (2026)	China; six medical institutions in Xuzhou	Clinical nurses with adverse-event history; n = 422	Analytical cross-sectional study	Organizational and institutional support; Safety culture, psychological safety, communication openness, and organizational learning	Second-victim distress and recovery	10.1186/s12912-026-04515-0

Abbreviations: CRNA, certified registered nurse anesthetist; HCP, healthcare professional; ICU, intensive care unit; NR, not reported; PECOS, Population, Exposure, Comparator, Outcomes, and Study design; PICU, pediatric intensive care unit; PSI, patient safety incident.
