# Supplementary material for: Leadership and Organizational Responses After Patient Safety Incidents: A Systematic Review of Second-Victim Outcomes and Evidence Gaps in Safety Behaviors
Source: Healthcare (Basel). 2026 Sep 14;14(18):3002. doi: 10.3390/healthcare14183002 (PMC13606040; doi:10.3390/healthcare14183002)
Supplement: Supplementary file 1 [file healthcare-14-03002-s001.zip › Supplementary Table S2.pdf]

Supplementary Table S2

Study-Level and Item-Level JBI Critical Appraisal of the 44 Included Studies

Leadership and Organizational Responses After Patient Safety Incidents: A Systematic Review of Second-Victim Outcomes and Evidence Gaps in Safety Behaviors

Appraisal approach. Design-specific critical appraisal was conducted using the JBI instrument appropriate to the eligible quantitative design of each study. The item-level judgments were subsequently reviewed and agreed by two reviewers, with any discrepancies resolved through discussion and consensus. Judgments were recorded as Yes (Y), No (N), Unclear (U), or Not applicable (NA). No composite score or numerical quality threshold was used, and studies were not excluded solely because of appraisal findings. “Unclear” indicates that the published report did not provide enough information to establish that the criterion was met; it does not necessarily indicate that the procedure was not performed.

Design allocation. The analytical cross-sectional checklist was applied to 34 studies, including the eligible cross-sectional quantitative component of the mixed-methods study and the cross-sectional outcome evaluation embedded in one program report. The quasi-experimental checklist was applied to nine uncontrolled pre-post, feasibility, quality-improvement, or program evaluations. The case-control checklist was applied to one retrospective case-control intervention evaluation.

Item key

Checklist	Item codes	Condensed appraisal criteria
Analytical cross-sectional	C1-C8	C1 clear inclusion criteria; C2 participants/setting described; C3 exposure measured validly/reliably; C4 objective/standard condition criteria; C5 confounders identified; C6 confounding addressed; C7 outcomes measured validly/reliably; C8 appropriate statistics.
Quasi-experimental	Q1-Q9	Q1 cause precedes effect; Q2 comparison participants similar; Q3 care similar apart from intervention; Q4 control group; Q5 multiple pre- and post-intervention measurements; Q6 follow-up complete/handled; Q7 outcomes measured consistently; Q8 reliable outcome measurement; Q9 appropriate statistics.
Case-control	CC1-CC10	CC1 groups otherwise comparable; CC2 appropriate matching; CC3 same identification criteria; CC4 valid/reliable exposure measurement; CC5 exposure measured identically; CC6 confounders identified; CC7 confounding addressed; CC8 outcomes measured validly/reliably and identically; CC9 meaningful exposure period; CC10 appropriate statistics.

The condensed item labels above paraphrase the JBI checklist criteria for table readability. The appraisal followed the design-specific JBI instruments cited in the main manuscript [54].

Summary of appraisal findings

Across the cross-sectional evidence, reporting of eligibility, setting, measurement, and statistical methods was generally adequate. The dominant limitation was inherent temporal ambiguity from concurrent measurement, together with self-report, convenience or voluntary recruitment, and potential residual confounding. The quasi-experimental evidence was consistently limited by the absence of concurrent control groups and by lack of multiple measurements before and after implementation. The single case-control evaluation used non-contemporaneous groups, increasing susceptibility to selection and secular confounding. These concerns were incorporated into the outcome-specific narrative interpretation and informed the domain-specific narrative confidence statements and SWiM-informed summary presented in Table 6 of the revised main manuscript.

Analytical cross-sectional checklist (n = 34)

Judgment	C1	C2	C3	C4	C5	C6	C7	C8
Y	33	32	30	31	30	30	33	34
N	0	0	0	0	4	4	0	0
U	1	2	4	3	0	0	1	0
NA	0	0	0	0	0	0	0	0

Quasi-experimental checklist (n = 9)

Judgment	Q1	Q2	Q3	Q4	Q5	Q6	Q7	Q8	Q9
Y	9	3	0	0	0	2	7	4	7
N	0	0	0	9	9	2	0	0	0
U	0	6	9	0	0	5	2	5	2

Judgment	Q1	Q2	Q3	Q4	Q5	Q6	Q7	Q8	Q9
NA	0	0	0	0	0	0	0	0	0

Case-control checklist (n = 1)

Judgment	CC1	CC2	CC3	CC4	CC5	CC6	CC7	CC8	CC9	CC10
Y	0	0	1	1	1	1	0	1	1	1
N	1	1	0	0	0	0	0	0	0	0
U	0	0	0	0	0	0	1	0	0	0
NA	0	0	0	0	0	0	0	0	0	0

Table S2A. JBI analytical cross-sectional appraisal (n = 34)

Study	Author/year	C1	C2	C3	C4	C5	C6	C7	C8	Principal appraisal considerations
S01 [30]	Burlison et al. (2021)	Y	Y	Y	Y	Y	Y	Y	Y	Cross-sectional self-report and concurrent mediation analysis; temporal ordering cannot be established.
S03 [57]	Nijs et al. (2021)	Y	Y	U	U	N	N	U	Y	Custom support/culture ratings and bivariate associations; no confounder-adjusted analysis.
S04 [20]	Choi et al. (2022)	Y	Y	Y	Y	Y	Y	Y	Y	Online cross-sectional latent profiles; self-selection and concurrent measurement limit causal interpretation.
S05 [58]	Eslami et al. (2022)	Y	Y	Y	Y	N	N	Y	Y	Convenience cross-sectional sample and correlation-based analysis without confounder adjustment.
S06 [59]	Gupta et al. (2022)	Y	U	U	U	Y	Y	Y	Y	Online professional-network survey; sampling frame and exposure classification were incompletely reported.
S07 [60]	Hinkley et al. (2022)	Y	Y	Y	Y	Y	Y	Y	Y	Cross-sectional structural model using self-reported constructs; model direction does not demonstrate temporality.
S08 [61]	Huang et al. (2022)	Y	Y	Y	Y	Y	Y	Y	Y	Large cross-sectional self-report study; residual confounding and common-method bias remain possible.
S09 [62]	Kim et al. (2022)	Y	Y	Y	Y	Y	Y	Y	Y	Cross-sectional modeling across hospital departments; concurrent measurement limits temporal inference.
S10 [19]	Mallea Salazar et al. (2022)	Y	Y	Y	Y	N	N	Y	Y	Non-probability convenience sampling and principally unadjusted correlational/contingency analyses.
S11 [25]	Mohd Kamaruzaman et al. (2022)	Y	Y	Y	Y	Y	Y	Y	Y	Multicentre mediation analysis, but all exposure, mediator, and outcome variables were measured concurrently.

Study	Author/year	C1	C2	C3	C4	C5	C6	C7	C8	Principal appraisal considerations
S16 [31]	Shao et al. (2023)	Y	Y	Y	Y	Y	Y	Y	Y	Convenience sample and latent-profile classification; associations remain sample-dependent and cross-sectional.
S17 [22]	Sun et al. (2023)	Y	Y	Y	Y	Y	Y	Y	Y	Cross-sectional self-report; turnover intention rather than observed turnover and possible residual confounding.
S21 [21]	Scarpis et al. (2024)	Y	Y	Y	Y	Y	Y	Y	Y	Only the adverse-event-exposed subgroup informed eligible analyses; latent profiles and outcomes were concurrent.
S22 [68]	Bexten et al. (2025)	Y	Y	Y	Y	Y	Y	Y	Y	Symposium-based convenience recruitment and retrospective self-report may introduce selection and recall bias.
S25 [70]	Fall et al. (2025)	U	U	U	U	N	N	Y	Y	Program-offer exposure was non-randomized and survey denominators were incompletely reported; selection/referral bias likely.
S26 [33]	Hakamy et al. (2025)	Y	Y	Y	Y	Y	Y	Y	Y	Small cross-sectional sample; self-reported turnover intention and limited precision/generalizability.
S27 [71]	Istrate et al. (2025)	Y	Y	Y	Y	Y	Y	Y	Y	Mixed online/onsite cross-sectional recruitment; self-report and residual confounding remain possible.
S28 [24]	Jeong et al. (2025)	Y	Y	Y	Y	Y	Y	Y	Y	Path analysis was based on concurrently measured self-report variables; causal sequencing cannot be confirmed.
S29 [45]	Kim et al. (2025)	Y	Y	Y	Y	Y	Y	Y	Y	PLS-SEM associations used self-reported practice changes and cannot establish temporal ordering.
S30 [23]	Lee and Lee (2025)	Y	Y	Y	Y	Y	Y	Y	Y	Adjusted serial mediation was cross-sectional; reverse and alternative pathways remain possible.
S31 [72]	Liu et al. (2025)	Y	Y	Y	Y	Y	Y	Y	Y	Latent classes are sample-dependent; all measures were self-reported and measured at one time point.
S32 [27]	Mahat et al. (2025)	Y	Y	Y	Y	Y	Y	Y	Y	Small cross-sectional mediation sample; intentions/absence were self-reported and temporal order was unverified.
S33 [74]	Rai et al. (2025)	Y	Y	U	Y	Y	Y	Y	Y	International voluntary online survey; support exposure measurement and non-response represent possible bias.

Supplementary Table S2 - JBI Critical Appraisal

Study	Author/year	C1	C2	C3	C4	C5	C6	C7	C8	Principal appraisal considerations
S34 [15]	Shin et al. (2025)	Y	Y	Y	Y	Y	Y	Y	Y	Cross-sectional relationships among distress, support, collaboration, and growth cannot establish directionality.
S35 [28]	Zheng et al. (2025)	Y	Y	Y	Y	Y	Y	Y	Y	Convenience sample from three tertiary Grade A hospitals; self-reported correlates limit external validity and causal inference.
S36 [73]	Qiu et al. (2026)	Y	Y	Y	Y	Y	Y	Y	Y	Cross-sectional mediation after patient suicide; exposure, mediator, and burnout were measured concurrently.
S37 [75]	Aikawa et al. (2026)	Y	Y	Y	Y	Y	Y	Y	Y	National voluntary survey with low availability of formal support; self-selection and cross-sectional mediation.
S38 [76]	Heo et al. (2026)	Y	Y	Y	Y	Y	Y	Y	Y	Online cross-sectional survey with retrospective six-month incident recall; residual confounding remains possible.
S39 [77]	Kang and Kim (2026)	Y	Y	Y	Y	Y	Y	Y	Y	Single-hospital convenience sample; all constructs and turnover intention were self-reported concurrently.
S40 [78]	Kappes et al. (2026)	Y	Y	Y	Y	Y	Y	Y	Y	Multinational convenience survey; country/system differences and self-reported exposure/outcomes may confound associations.
S41 [17]	Long et al. (2026)	Y	Y	Y	Y	Y	Y	Y	Y	Cross-sectional design and retrospective self-evaluation of prior events; regional sample limits generalizability.
S42 [79]	Ren et al. (2026)	Y	Y	Y	Y	Y	Y	Y	Y	Quantitative component was cross-sectional despite mixed-methods design; actual turnover was not observed.
S43 [16]	Siavashi et al. (2026)	Y	Y	Y	Y	Y	Y	Y	Y	Cross-sectional descriptive design with concurrent validated measures; temporal direction cannot be established.
S44 [80]	Zong et al. (2026)	Y	Y	Y	Y	Y	Y	Y	Y	Convenience multicentre cross-sectional mediation; common-method bias and unverified temporal ordering.

Table S2B. JBI quasi-experimental appraisal (n = 9)

Study	Author/year	Q1	Q2	Q3	Q4	Q5	Q6	Q7	Q8	Q9	Principal appraisal considerations
S02 [36]	Finney et al. (2021)	Y	U	U	N	N	U	Y	Y	Y	Independent pre/post survey samples, no concurrent control, and possible differences in respondents over time.
S12 [37]	Thompson et al. (2022)	Y	U	U	N	N	U	U	U	U	Independent pre/post samples, short one-month follow-up, no concurrent control, and no statistically significant change in SVEST scores.
S13 [63]	Turner et al. (2022)	Y	U	U	N	N	U	U	U	Y	Uncontrolled service-level before/after evaluation; pre-implementation process audit was incomplete and secular effects possible.
S14 [64]	Li et al. (2023)	Y	Y	U	N	N	Y	Y	Y	Y	Single-group repeated-measures intervention with no control; one baseline and short eight-week follow-up.
S15 [39]	Pelikan et al. (2023)	Y	U	U	N	N	U	Y	Y	Y	Unmatched pre/post respondents over two years; exposure to and use of the program were not uniform.
S18 [65]	Webb et al. (2023)	Y	U	U	N	N	N	Y	U	U	Very small post-implementation sample, COVID-19 period effects, and incomplete documentation of peer-support encounters.
S19 [66]	Bursch et al. (2024)	Y	U	U	N	N	U	Y	U	Y	Repeated cross-sectional surveys without control; small event-exposed subset and wide confidence intervals.
S20 [67]	Choi et al. (2024)	Y	Y	U	N	N	N	Y	Y	Y	Single-group feasibility study with 10 paired participants and substantial attrition from applicants.
S24 [42]	Davis et al. (2025)	Y	Y	U	N	N	Y	Y	U	Y	Immediate pre/post evaluation of 13 encounters; no control, convenience sample, and no longer-term follow-up.

Table S2C. JBI case-control appraisal (n = 1)

Study	Author/year	CC1	CC2	CC3	CC4	CC5	CC6	CC7	CC8	CC9	CC10	Principal appraisal considerations
S23 [69]	Chen et al. (2025)	N	N	Y	Y	Y	Y	U	Y	Y	Y	Retrospective non-contemporaneous comparison (2022 vs. 2023); selection, secular change, and residual confounding limit attribution.

Abbreviations: Y, yes; N, no; U, unclear because of insufficient reporting; NA, not applicable. Item codes are defined in the item key. No total score was calculated.

Interpretive note. Appraisal judgments reflect the information reported in each publication and its available supplementary material. Study-specific concerns were considered in the structured narrative synthesis and directly informed the domain-specific narrative confidence interpretation presented in Sections 3.7-3.8 and Table 6 of the revised manuscript. In particular, statistically modeled indirect effects from cross-sectional data were treated as associational rather than causal, and implementation outcomes were not interpreted as evidence of improved safety behavior or patient outcomes unless those outcomes were directly measured.
