# Supplementary material for: Leadership and Organizational Responses After Patient Safety Incidents: A Systematic Review of Second-Victim Outcomes and Evidence Gaps in Safety Behaviors
Source: Healthcare (Basel). 2026 Sep 14;14(18):3002. doi: 10.3390/healthcare14183002 (PMC13606040; doi:10.3390/healthcare14183002)
Supplement: Supplementary file 1 [file healthcare-14-03002-s001.zip › Supplementary Table S3.pdf]

Supplementary Table S3

Reports Excluded After Full-Text Assessment, with Primary Reasons for Exclusion

Leadership and Organizational Responses After Patient Safety Incidents: A Systematic Review of Second-Victim Outcomes and Evidence Gaps in Safety Behaviors

Note. One primary reason was assigned to each report excluded after full-text assessment. Category totals correspond to the PRISMA flow diagram: no eligible leadership-related or organizational exposure-outcome analysis (n = 14); wrong population or no separable incident-affected healthcare-professional subgroup (n = 4); and implementation-only evaluation without an eligible prespecified post-incident outcome (n = 3).

A. No eligible leadership-related or organizational exposure-outcome analysis (n = 14)

No.	Report	Specific reason for exclusion
1	Finney, R.E.; Torbenson, V.E.; Riggan, K.A.; et al. Second victim experiences of nurses in obstetrics and gynaecology: A Second Victim Experience and Support Tool survey. J. Nurs. Manag. 2021, 29, 642-652. doi:10.1111/jonm.13198.	Described second-victim experiences and perceived support resources but did not quantitatively test an association between a predefined leadership-related or organizational exposure and an eligible post-incident outcome.
2	Prametz, R.; Koch, P.; Vogelgesang, A.; et al. Prevalence of second victims, risk factors and support strategies among young German physicians in internal medicine (SeViD-I survey). J. Occup. Med. Toxicol. 2021, 16, 11. doi:10.1186/s12995-021-00300-8.	Estimated prevalence, individual risk factors, and preferred support strategies; no eligible leadership-related or organizational exposure-outcome analysis was reported.
3	Santana-Dominguez, I.; Gonzalez-de la Torre, H.; Verdu-Soriano, J.; et al. Feelings of being a second victim among Spanish midwives and obstetricians. Nurs. Open 2022, 9, 2356-2369. doi:10.1002/nop2.1249.	Characterized second-victim feelings and experiences without quantitatively relating an eligible leadership-related or organizational exposure to a prespecified outcome.
4	Allender, E.A.; Bottema, S.M.; Bosley, C.L.; et al. Use of the Revised Second Victim Experience and Support Tool to examine second victim experiences of respiratory therapists. Respir. Care 2023, 68, 749-759. doi:10.4187/respcare.10719.	Used the revised SVEST to describe experiences and support perceptions; no eligible organizational exposure-outcome relationship was evaluated.
5	Lim, S.H.; Zainal, H.; Lee, L.J.; et al. Second victim experiences and impact among acute care nurses: An exploratory study. Int. Nurs. Rev. 2025, 72, e12999. doi:10.1111/inr.12999.	Explored second-victim experiences and impacts but did not assess an eligible leadership-related or organizational exposure linked quantitatively to an eligible outcome.
6	Alishaq, M.; Hassan, A.K.; Assar, A.H.; et al. The impact of second-victim syndrome on staff well-being and practice. Medicine 2025, 104, e41345. doi:10.1097/MD.0000000000041345.	Examined the relationship of second-victim syndrome with staff well-being and practice, but no eligible leadership-related or organizational exposure was assessed.
7	Xie, N.; Ye, L.; Tan, P. Second victim experiences and perceived support among newly registered nurses following patient safety incidents: A cross-sectional study. Front. Public Health 2026, 14, 1813803. doi:10.3389/fpubh.2026.1813803.	Described second-victim experiences and perceived support; the report did not provide an eligible quantitative leadership/organizational exposure-outcome analysis.
8	Bushuven, S.; Taubner, T.P.; Trifunovic-Koenig, M.; et al. Presence and detectability of the second victim phenomenon in German-speaking medical professionals involved in dysphagia and respiratory therapy: Cross-sectional survey research (SeViD-V). BMC Health Serv. Res. 2026, 26, 809. doi:10.1186/s12913-026-14648-2.	Focused on prevalence, detectability, and support strategies; no eligible leadership-related or organizational exposure-outcome association was evaluated.
9	Quintano, L.; Vella Bonanno, P.; Strametz, R.; Klemm, V.; Buttigieg, S.C. The second victim phenomenon in medical practitioners, nurses, and pharmacists: The SeViMa study for Malta. J. Public Health 2026, advance online publication. doi:10.1007/s10389-026-02825-w.	Described the occurrence and characteristics of the second-victim phenomenon across professions without an eligible quantitative organizational exposure-outcome analysis.
10	Brunelli, M.V.; Estrada, S.; Celano, C.; et al. Segunda víctima: Experiencia y medidas de apoyo percibidas por los profesionales sanitarios. Medicina (B. Aires) 2023, 83, 918-926.	Reported second-victim experiences and perceived support measures descriptively; no eligible leadership-related or organizational exposure-outcome analysis was presented.
11	Alfar, Z.; Hakamy, E.; Innab, A. The impacts of second victim experiences on nurses' absenteeism and intention to leave: A multi-site cross-sectional study. J. Adv. Nurs. 2025, 81, 4817-4827. doi:10.1111/jan.16695.	Companion report based on the same 117-nurse multisite sample as Hakamy et al. [33]; it did not provide a distinct eligible leadership/organizational exposure-outcome analysis and was not counted as a separate eligible study.
12	Xu, Y.; Jin, Q.; Zhou, Q.; Zhang, R.; Ding, W. Relationship between second victim experience and turnover intention in Chinese nurses: Assessing the mediating role of posttraumatic growth using structural equation modeling. J. Nurs. Manag. 2025, 2025, 7327139. doi:10.1155/jonm/7327139.	Modeled second-victim experience, post-traumatic growth, and turnover intention without an eligible leadership-related or organizational exposure.
13	Al Sabei, S.; Qutishat, M. Second victim syndrome and turnover intention among critical care nurses. Discov. Ment. Health 2025, 5, 110. doi:10.1007/s44192-025-00256-9.	Assessed second-victim syndrome in relation to turnover intention but did not include an eligible leadership-related or organizational exposure.
14	Neyens, L.; Stouten, E.; Vanhaecht, K.; et al. Open disclosure among general practitioners as second victim of a patient safety incident: A cross-sectional study in Flanders (Belgium). J. Patient Saf. 2025, 21, 9-14. doi:10.1097/PTS.0000000000001299.	Open disclosure was assessed, but the report did not quantitatively link disclosure to an eligible leadership-related or organizational exposure.

B. Wrong population or no separable subgroup of healthcare professionals affected by a patient safety incident (n = 4)

No.	Report	Specific reason for exclusion
15	Sexton, J.B.; Adair, K.C.; Profit, J.; et al. Perceptions of institutional support for “second victims” are associated with safety culture and workforce well-being. Jt. Comm. J. Qual. Patient Saf. 2021, 47, 306-312. doi:10.1016/j.jcjq.2020.12.001.	The survey sampled the general healthcare workforce and assessed perceptions of support for affected colleagues; it did not restrict analyses to professionals personally affected by a patient safety incident or provide a separable eligible subgroup.

No.	Report	Specific reason for exclusion
16	Fisher, S.; Djerassi, R.; Gendler, Y.; Pirogovsky, A.; Dreiher, J. Uncovering unmet needs in healthcare teams: A cross-sectional study on the second victim phenomenon. Isr. J. Health Policy Res. 2026, 15, 19. doi:10.1186/s13584-026-00764-1.	The survey was distributed to all physicians and nurses rather than a confirmed incident-affected cohort; no separable subgroup of healthcare professionals affected by a patient safety incident was analyzed for the eligible exposure-outcome relationship.
17	High, A.E.; Forest, S. Peer support targeting the second victim phenomenon: Implementation and outcomes. Cureus 2025, 17, e78854. doi:10.7759/cureus.78854.	Department-wide pre/post surveys and support encounters included both adverse events and non-patient-safety stressors; an incident-affected healthcare-professional subgroup was not separately analyzed for eligible outcomes.
18	McCarthy, S.E.; Finn, M.; Valentelyte, G.; et al. Implementing after action review in an Irish tertiary hospital: A mixed-methods evaluation of safety culture and second victim experience and support. BMC Health Serv. Res. 2026, advance online publication. doi:10.1186/s12913-026-15134-5.	Hospital-wide repeated surveys included all staff and did not provide a separable subgroup of healthcare professionals personally affected by a patient safety incident for the eligible exposure-outcome analysis.

C. Implementation-only evaluation without an eligible prespecified post-incident outcome (n = 3)

No.	Report	Specific reason for exclusion
19	Schroder, K.; Bovil, T.; Jorgensen, J.S.; Abrahamsen, C. Evaluation of “the Buddy Study”, a peer support program for second victims in healthcare: A survey in two Danish hospital departments. BMC Health Serv. Res. 2022, 22, 566. doi:10.1186/s12913-022-07973-9.	The evaluation focused on seminar satisfaction, perceived usefulness, program experience, and qualitative implementation findings; it did not measure an eligible prespecified post-incident outcome linked to the intervention.
20	Rivera-Chiauzzi, E.Y.; Huang, L.; Osborne, A.K.; et al. Rapid expansion of the Healing Emotional Lives of Peers program during COVID-19: A second victim peer support program for healthcare professionals. J. Patient Saf. 2024, 20, 28-37. doi:10.1097/PTS.0000000000001179.	Reported training, program activations, peer-support helpfulness, and participant satisfaction; these were implementation/acceptability outcomes rather than an eligible prespecified post-incident outcome.
21	Groves, K.A.; Lewis, A.; Hasson, D.C.; Haines, E. Implementation of an institutional physician and advanced practicing provider peer support program. J. Patient Saf. 2026, advance online publication. doi:10.1097/PTS.0000000000001528.	Described program establishment, peer-supporter training, referral volume, utilization, and reasons for referral without measuring an eligible prespecified post-incident outcome.
